# Optineurin binding to the novel interacting partner Junction plakoglobin prevents muscle atrophy in mice

**DOI:** 10.1371/journal.pbio.3003581

**Published:** 2026-01-22

**Authors:** Xiao Chen Shi, Rui Xin Zhang, Jun Kai Feng, Jia Hao Chen, Jian Feng Zhang, Jun Ying Xiao, Xiao Peng Liu, Huan Liu, Bo Xia, Li Nong Yao, Jiang Wei Wu

**Affiliations:** 1 Key Laboratory of Animal Genetics, Breeding and Reproduction of Shaanxi Province, College of Animal Science and Technology, Northwest A&F University, Yangling, Shaanxi, China; 2 Department of Pharmacology, School of Basic Medical Science, Shanxi Medical University, Taiyuan, Shanxi, China; 3 Department of Emergency Medicine, the 942nd Hospital of PLA Joint Logistics Support Force, Yinchuan, Ningxia, China; 4 Department of Critical Care Medicine, Tangdu Hospital, Air Force Medical University, Xi’ an, Shaanxi, China; King’s College London, UNITED KINGDOM OF GREAT BRITAIN AND NORTHERN IRELAND

## Abstract

Skeletal muscle atrophy is a debilitating condition that significantly affects patients’ quality of life and prognosis, yet its underlying mechanisms remain poorly understood. Here, we identify Optineurin (OPTN) as an active regulator for maintenance of muscle homeostasis during muscle atrophy. Knockdown (KD) of *Optn* induces muscle atrophy, while overexpression of *Optn* alleviated dexamethasone-induced muscle atrophy in mice. Mechanistically, we for the first time identified Junction plakoglobin (JUP) as a novel interacting partner of OPTN. OPTN alleviates muscle atrophy in a JUP-dependent manner, corroborating JUP as the downstream effector of OPTN-mediated muscle atrophy. RNA-seq analysis revealed that PI3K-AKT pathway is markedly downregulated in *Optn*-KD muscle, and pharmacological activation of PI3K-AKT pathway effectively rescued muscle atrophy in *Optn*-KD mice. We further show that OPTN coordinates the interaction between JUP and PI3-Kinase p85 in muscle, promoting activation of the PI3K-AKT pathway. Collectively, our study proposed a conceptual novelty that OPTN-JUP axis mediated activation of the PI3K-AKT pathway during muscle atrophy. These findings offer new insights into the mechanisms of muscle atrophy and suggest potential therapeutic strategies for this condition.

## Abstract 2

Skeletal muscle atrophy is a debilitating condition that accompanies a number of diseases, but there are few known therapeutic targets to reverse it. This study identifies Optineurin as a regulator of muscle homeostasis, showing that it binds to JUP and activates the PI3K-AKT pathway to prevent muscle atrophy in mice.

## Introduction

Skeletal muscle atrophy is a clinical manifestation of muscle loss due to wasting and catabolism caused by disuse related to a sedentary lifestyle and lack of physical activity, as well as to conditions such as intensive care unit-acquired weakness, cancer cachexia, Duchenne Muscular Dystrophy, and aging-related diseases [1,2]. A major contributor to chronic stress-induced muscle wasting is elevated serum levels of glucocorticoids (GCs) [3,4], an important regulatory hormone in humans. In muscle cells, GCs inhibit protein synthesis by inhibiting the stimulatory action of insulin or insulin-like growth factor on muscle protein synthesis [5]. Apart from inhibiting the rate of muscle protein synthesis, muscle atrophy induces protein degradation via activating the nuclear translocation of muscle atrophy-related transcription factors (FoxO transcriptional factor) [6]. Although muscle atrophy has been extensively studied, the molecular regulators capable of modulating muscle atrophy remain poorly understood. In addition to exercise intervention, there are few effective therapeutic strategies and pharmacological targets for treatment of muscle atrophy [7]. Therefore, development of improved approaches or novel target discovery will require elucidation of the regulatory mechanisms governing muscle atrophy.

Optineurin (OPTN) is a cytosolic protein containing 577 amino acid residues, which is highly expressed in skeletal muscle [8]. Mutations in *OPTN* are associated with various degenerative diseases, such as amyotrophic lateral sclerosis (ALS) [9], indicating that OPTN is involved in muscle function. We previously showed that OPTN plays an essential role in myogenesis during muscle regeneration [10], and Ishikawa and colleagues also found that *Optn* knockdown inhibits myogenic differentiation by downregulation of myogenin and myoblast determination protein in C2C12 myoblasts [11]. These studies suggest a significant implication of OPTN in functions of muscle development and regeneration. Besides, *OPTN* mutation is also associated with other human diseases, such as glaucoma and Paget’s disease of bone. Mechanistically, interacting with various proteins, OPTN is a critical factor of multiple basic cellular processes and events, including vesicle trafficking, maintenance of the Golgi apparatus, and autophagy [12]. The previous study showed that OPTN localizes in vesicles at the plasma membrane, where it can bind to myosin VI and RAB8a, involved in endocytosis and vesicle trafficking [13]. It has also been identified as a selective autophagy receptor involved in the various stages of the autophagic process, such as cargo recognition, autophagosome formation, and autophagic degradation [12]. Our previous study showed that OPTN activates Wnt signaling pathway through physically interacting and targeting GSK3β for autophagic degradation in C2C12 myoblasts [10], suggesting the function of OPTN upon autophagy in muscle. However, whether OPTN also plays a pivotal role in muscle atrophy and its underlying mechanism is completely unknown.

Growth of skeletal muscle, like that of dividing cells, is largely dependent on signaling through the PI3K-AKT signaling pathway. It has been reported that the PI3K-AKT signaling pathway is important during GC-induced skeletal muscle atrophy, in which GC inhibits PI3K activity through promoting interaction with GR and the PI3K regulatory subunit p85, inhibiting the binding of p85 to insulin receptor substrate-1 [14]. The reduced activity of this pathway promotes the nuclear translocation of FoxO transcriptional factors [15]. The FoxO transcription factors play a major role in muscle atrophy by inducing the expression of atrogenes, which include specialized E3 ubiquitin ligases such as Atrogin-1/muscle atrophy F-box protein and muscle RING finger-1 protein (MuRF-1) [16]. Junction plakoglobin (JUP) is a component of desmosome adhesion complexes that are prominent in tissues that must withstand mechanical stress, and it also controls cell motility, growth, and differentiation through mediating various signaling pathways in epithelia [17]. Recently, JUP was identified as a modulator of insulin receptor activity, and overexpression of JUP enhances the PI3K-AKT signaling pathway and promotes muscle growth in mice [18]. Mechanistically, JUP activates the PI3K-AKT signaling pathway through binding to insulin receptor and the PI3K subunit p85 in muscle, which has a process of translocation from cytoplasm to cell membrane [19]. Nevertheless, the underlying mechanism of JUP translocation from cytoplasm to cell membrane remains incompletely resolved in muscle [19], and whether it plays an important role in muscle atrophy remains unknown.

In this study, we show that OPTN is required for the maintenance of skeletal muscle homeostasis during muscle atrophy. We demonstrate that OPTN activates the PI3K-AKT signaling pathway through coordinating the association of PI3 Kinase p85 and JUP during muscle atrophy. Our findings reveal a novel insight into mechanism underlying muscle atrophy and provide a potential therapeutic target for its treatment.

## Results

### OPTN is down-regulated in muscle atrophy

**Fig 1:**
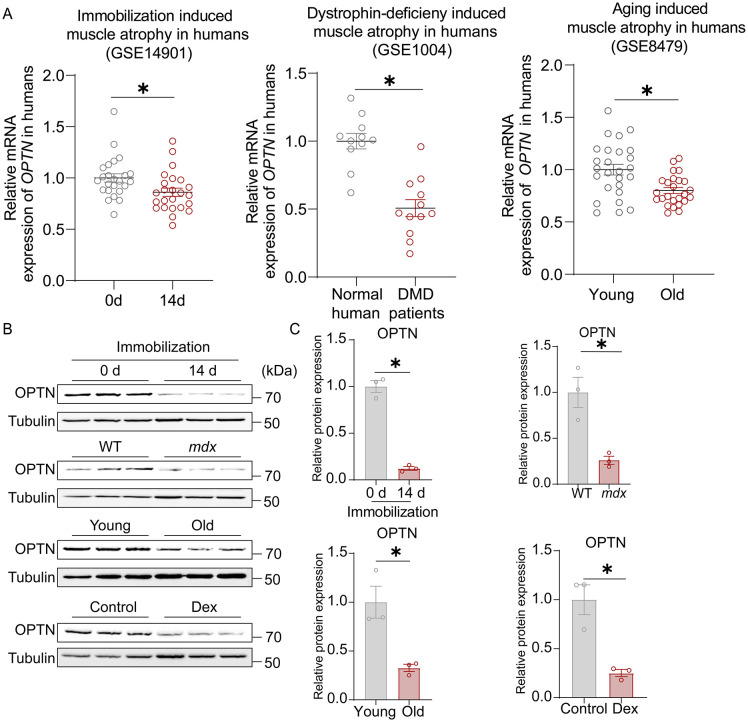
OPTN is down-regulated in muscle atrophy. **(A)** The relative *OPTN* mRNA expression in human skeletal muscle of patients with different types of muscular atrophy, including immobilization (for control sample taken at time 0 d, *n* = 24 biological independent samples; for immobilization sample taken at time 14 d from the limb was immobilized via a brace, *n* = 24 biological independent samples), dystrophin-deficiency (for normal human skeletal muscle sample, *n* = 12 biological independent samples; for Duchenne Muscular Dystrophy patient’s skeletal muscle sample, *n* = 12 biological independent samples), and aging-induced muscular atrophy [Skeletal muscle biopsies from older (*n* = 25 biological independent samples) and younger (*n* = 26 biological independent samples)]. **(B, C)** Representative immunoblotting analysis (B) and quantification (C) of OPTN in TA of different muscle atrophy models (immobilization, dystrophin-deficiency, and aging-induced muscle atrophy, as well as Dex-induced muscle atrophy) in mice (*n* = 3 mice in each group). Data are presented as mean ± standard error of the mean (SEM). ^*^P < 0.05 vs. control. The underlying data for this figure can be found in S1 Data. The Original blot for this figure can be found in S1 Raw Image.

We previously reported that OPTN is a novel regulator of myogenesis [10]. In this study, we asked whether OPTN plays an active role in controlling muscle growth and can cause muscle atrophy when defective. In the publicly available GEO database, we found that *Optn* expression was significantly reduced in skeletal muscle of patients with different types of muscular atrophy, including immobilization, dystrophin-deficiency, and aging-induced muscular atrophy (*P* < 0.05) (Fig 1A). To further explore the association between OPTN and muscle atrophy in mice, we measured mRNA and protein levels of OPTN in various types of muscle atrophy and found dramatically decreased levels in the skeletal muscle of mice with physical immobilization, dystrophin-deficiency, aging and dexamethasone (Dex)-induced muscle atrophy models (*P* < 0.05) (Figs 1B, 1C, and S1A–S1D). These results indicate a strong inverse association between *Optn* expression and muscle atrophy.

### *Optn* knockdown induces muscle atrophy in mice

**Fig 2:**
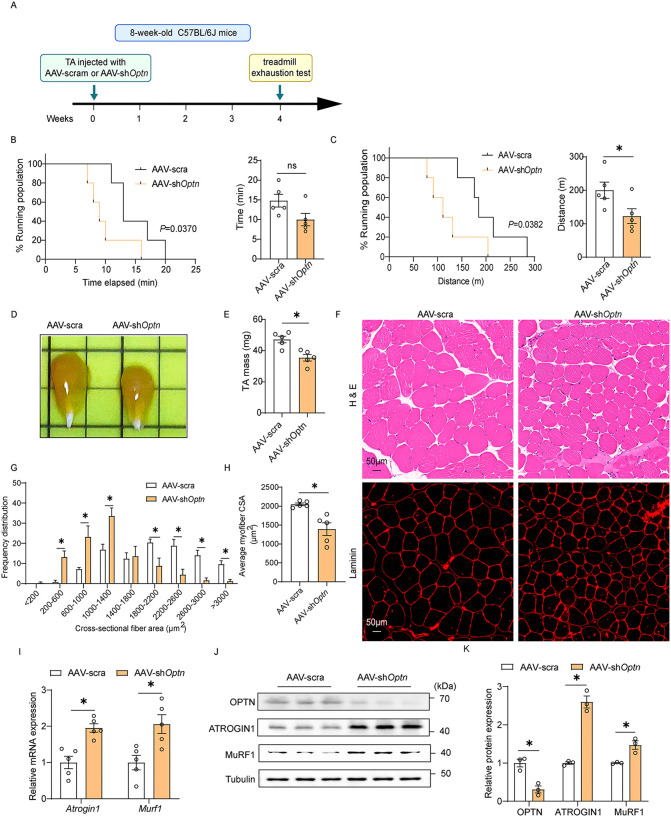
*Optn* knockdown induces muscle atrophy in mice. **(A)** Schematic representation of mouse model using AAV system to achieve *Optn* knockdown in TA muscle of 8-week-old mice for 4 weeks. **(B)** Time to exhaustion (left panel; survival plot showing the percentage of mice running at indicated time points. Right panel; mean duration of the run) (*n* = 5 mice in each group). **(C)** Running distance (left panel; survival plot showing the percentage of mice running at indicated distances. Right panel; mean distance ran) (*n* = 5 mice in each group). **(D)** Representative samples of dissected TA muscle in scramble shRNA or sh*Optn* mice. **(E)** The TA muscle mass in scramble shRNA or sh*Optn* mice (*n* = 5 mice in each group). **(F)** Representative H&E and laminin staining of TA muscle in scramble shRNA or sh*Optn* mice. Scale bar: 50 μm. **(G, H)** Distribution (G) and average (H) of myofiber CSA in scramble shRNA or sh*Optn* TA muscle (*n* = 5 mice in each group). **(I)** Relative mRNA expression of muscle atrogene (*Atrogin*-1 and *Murf*-1) in scramble shRNA or sh*Optn* TA muscle (*n* = 5 mice in each group). **(J, K)** Representative immunoblotting analysis (J) and quantification (K) of muscle atrophy markers (Atrogin-1 and Murf-1) in scramble shRNA or sh*Optn* TA muscle (*n* = 3 mice in each group). Data are presented as mean ± standard error of the mean (SEM). ^*^P < 0.05 vs. control. The underlying data for this figure can be found in S1 Data. The Original blot for this figure can be found in S1 Raw Image.

To investigate whether decreased *Optn* induces muscle atrophy, we constructed an adeno-associated virus expressing a short hairpin RNA (shRNA) against *Optn* (AAV-sh*Optn*), which achieved 90% virus infection efficiency in tibialis anterior (TA) muscle (S2A Fig). Four weeks after intramuscular injection of AAV-sh*Optn* or AAV-scramble to TA muscle in mice (Fig 2A), we performed a treadmill exhaustion test to assess exercise capacity and endurance since muscle-atrophy is often associated with defective muscle-related functions [20]. The results showed that the time to exhaustion and the running distance covered by *Optn* KD mice were significantly shorter than their control counterparts (*P* < 0.05) (Fig 2B and 2C), suggesting that *Optn* KD impairs the muscle-related functions. We further found that *Optn* KD significantly reduced weight (Fig 2D and 2E) and fiber size of TA muscle (*P* < 0.05) (Fig 2F–2H). Additionally, H&E staining revealed mild pathological changes of muscle fibers in *Optn* KD mice, including some edema and rounded muscle fibers observed (Fig 2F). Consistent with the histomorphological changes, the mRNA and protein levels of muscle atrophy marker Atrogin-1 and MuRF-1 were significantly upregulated in TA muscle of *Optn* KD mice (Fig 2I–2K). These results corroborate that *Optn* knockdown induces muscle atrophy. Together, these data show that reduction of OPTN induces muscle atrophy in vivo, suggesting an essential role of OPTN on the maintenance of muscle mass and functions.

### *Optn* overexpression prevents Dex-induced muscle atrophy

**Fig 3:**
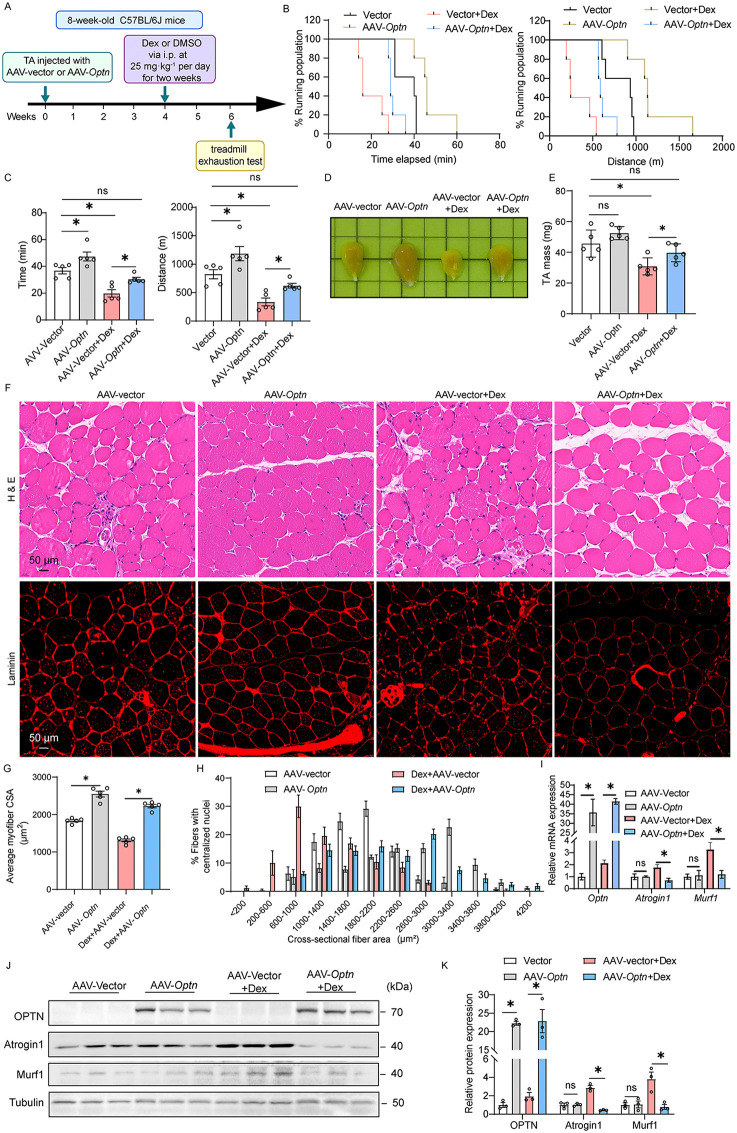
*Optn* overexpression protects against dexamethasone-induced muscle atrophy. **(A)** Schematics for restoring Dex-induced muscle atrophy by *Optn* overexpression in mice. Four weeks after intramuscular injection of AAV-*Optn* or AAV-vector to TA muscle in 8-week-old mice, the mice were then treated with Dex for muscular dystrophy induction for two weeks (Dex was administered intraperitoneally at 25 mg·kg^-1^ per day). **(B, C)** Physical performance was evaluated in mice by a treadmill exhaustion test (*n* = 5 mice in each group). Two parameters were measured with this test: (B) Time (Left panel) and Running distance (Right panel) to exhaustion (Survival plot showing the percentage of mice running at indicated time points and distances). (C) Quantification of mean duration (left panel) and distance of run to exhaustion (Right panel) (*n* = 5 mice in each group). **(D)** Comparison of representative samples of dissected TA muscle in control or *Optn*-overexpressing mice with Dex treatment. **(E)** The quantification of TA muscle mass in (D) (*n* = 5 mice in each group). **(F)** Representative H&E and laminin staining of TA muscle in control or *Optn*-overexpressing mice with Dex treatment (*n* = 5 mice in each group). Scale bar: 50 μm. **(G, H)** Average (G) and distribution (H) of myofiber CSAs in (F) (*n* = 5 mice in each group). **(I)** Relative mRNA expression of muscle atrogene (*Atrogin-1* and *Murf-1*) and *Optn* in control or *Optn*-overexpressing mice with Dex treatment (*n* = 3 mice in each group). **(J, K)** Representative immunoblotting analysis (I) and quantification (J) of muscle atrophy markers (Atrogin-1 and Murf-1) and OPTN in control or *Optn*-overexpressing mice with Dex treatment (*n* = 3 mice in each group). Data are presented as mean ± standard error of the mean (SEM). ^*^P < 0.05 vs. control. The underlying data for this figure can be found in S1 Data. The Original blot for this figure can be found in S1 Raw Image.

Having observed that *Optn* KD induces muscle atrophy, we then asked whether *optn* overexpression has therapeutic potential on the treatment of muscle atrophy. To this end, we constructed an adeno-associated virus expressing *Optn* (AAV-*Optn*), which achieved 90% virus infection efficiency in TA muscle (S2B Fig). Four weeks after intramuscular injection of AAV-*Optn* or AAV-vector to TA muscle, mice were then treated with Dex (Fig 3A), a commonly used synthetic GC for muscular dystrophy induction [21–24]. Compared with AAV-vector control group receiving Dex treatment, *Optn* overexpression markedly improved Dex-induced impairment of exercise capacity and endurance (Fig 3B, 3C). The mass and fiber size of TA muscle in *Optn-*overexpressing mice receiving Dex treatment were significantly larger than those in AAV-vector mice receiving Dex treatment (Fig 3D, 3E, and 3F–3H). Furthermore, *Optn* overexpression markedly improved Dex-induced pathological morphology of TA muscle with round fibers (Fig 3F). In line with this, the expression of Atrogin-1 and MuRF-1 in *Optn-*overexpressing mice receiving Dex treatment was remarkably decreased than those in AAV-vector mice receiving Dex treatment (Fig 3I–3K). These results showed that *Optn* overexpression protects against Dex-induced muscle atrophy.

### Identification of JUP as a novel interacting partner of OPTN in muscle atrophy

**Fig 4:**
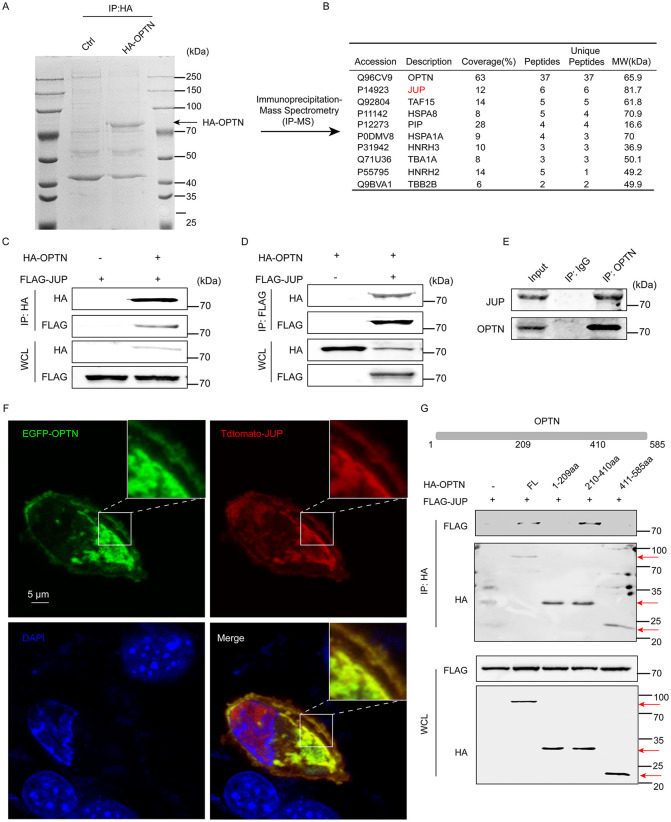
OPTN physically interacts with JUP in skeletal muscle. **(A, B)** A representative Coomassie staining of the HA-OPTN immune-complex isolated by immunoprecipitation analysis (A) and a list of novel OPTN-binding partners by mass spectrometry analysis (B). **(C, D)** Coimmunoprecipitation (Co-IP) assays were performed to examine the interaction of OPTN and JUP in HEK293T cells transfected with the indicated plasmids (*n* = 3 biologically independent samples). The immunoprecipitation analysis was performed in HEK293T cells incubated with anti-Flag or anti-HA magnetic beads. **(E)** Immunoprecipitation of endogenous OPTN and JUP in C2C12 cells (*n* = 3 biologically independent samples). The immunoprecipitation analysis was performed in C2C12 cells incubated with anti-OPTN antibody or nonspecific Rabbit IgG (control) to pulldown endogenous JUP. **(F)** Representative immunofluorescence analysis of EGFP-OPTN and Tdtomato-JUP in C2C12 cells transfected with EGFP-OPTN plasmids and Tdtomato-JUP plasmids (*n* = 3 biologically independent samples). Scale bars: 5 μm. **(G)** Schematic illustration of the OPTN full-length and fragments constructs, and the interaction domains of OPTN with JUP were explored based on immunoprecipitation analysis (*n* = 3 biologically independent samples). The Original blot for this figure can be found in S1 Raw Image.

To investigate underlying regulatory network of OPTN in muscle atrophy, we conducted immunoprecipitation and mass spectrometry analysis (IP-MS) to identify its potential interacting partners. HEK293T cells were used to overexpress HA-tagged OPTN. After precipitation with an anti-HA antibody, whole-cell lysates were eluted for in-gel MS assays (Fig 4A). MS analysis revealed the Top 10 novel OPTN-binding partners ranked by the number of unique peptides (Fig 4B). Among them, JUP stood out as a prominent and distinct binding partner, identified convincingly by six unique peptides, ranking first overall excepted OPTN in list (Fig 4B). We further validated this result in C2C12 cells and showed direct binding of OPTN with JUP by Co-IP (Fig 4C and 4D). We also showed the presence of JUP in the precipitate of endogenous OPTN in TA muscle by IP assay (Fig 4E), corroborating an endogenous binding of OPTN and JUP. Colocalization of ^EGFP^OPTN and ^Tdtomato^JUP in the cytoplasm and plasma membrane of C2C12 cells (Fig 4F) further supports their interaction. To identify the functional domains of OPTN that interact with JUP, a series of immunoprecipitation analyses upon OPTN truncation and JUP were performed. Our data mapped the 210−410 residues in OPTN as the major JUP-interaction region (Fig 4G). To explore the implication of OPTN–JUP interaction in muscle atrophy, we constructed the deletion mutant *Optn-*Δ210–410. Compared with wild type WT-*Optn*, *Optn-*Δ210–410 overexpression failed to rescue Dex-induced muscle atrophy in C2C12 cells (S3A and S3B Fig). In line with these morphological phenotypes, *Optn*-Δ210–410 overexpression was unable to restore down-regulated levels of muscle atrophy-related markers (MuRf-1 and Atrogin-1) in Dex-treated C2C12 cells compared with WT-*Optn* overexpression (S3C and S3D Fig).

### OPTN alleviates Dex-induced muscle atrophy in a JUP-dependent manner

**Fig 5:**
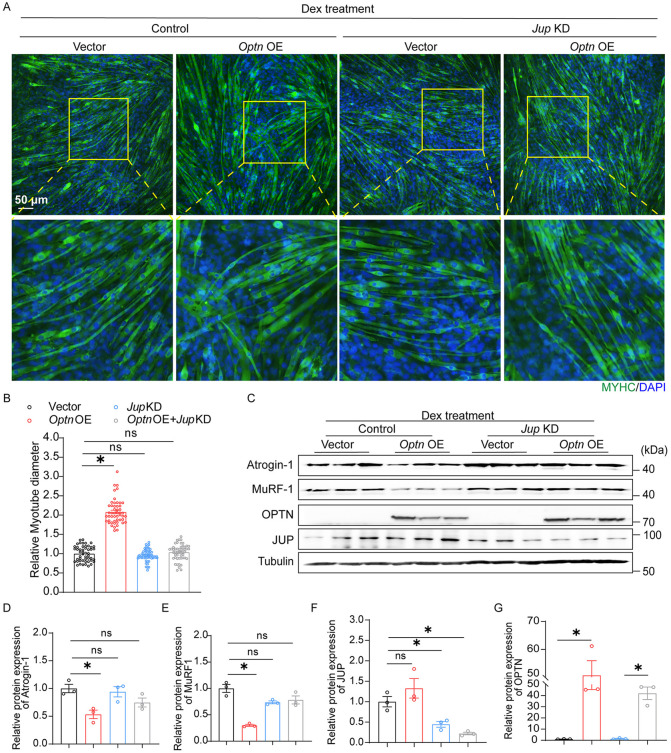
OPTN attenuates Dex-induced muscle atrophy in a JUP-dependent manner. **(A)** Representative immunofluorescence analysis of myotubes stained with MYHC (green) in *Optn* OE C2C12 cells with or without *Jup* KD at 4 d post-differentiation with Dex treatment (*n* = 3 biologically independent samples). Scale bar: 50 µm. **(B)** Quantification of fiber diameter in myotubes is described in (A) (*n* = 50 in each group). **(C–G)** Representative immunoblotting analysis (C) and quantification (D–G) of muscle atrophy markers (Atrogin-1 and Murf-1) (D, E), JUP (F), and OPTN (G) in *Optn* OE C2C12 cells with or without *Jup* KD at 4 d post-differentiation with Dex treatment (*n* = 3 in each group). Data are presented as mean ± standard error of the mean (SEM). ^*^P < 0.05 vs. control. The underlying data for this figure can be found in S1 Data. The Original blot for this figure can be found in S1 Raw Image.

Finally, we tested whether JUP is required for the protective effect of OPTN against Dex-induced muscle atrophy. In C2C12 cells, knockdown of *Jup* completely abolished the protective effect of OPTN overexpression on Dex-induced reduction in myotube diameter (Fig 5A and 5B). Consistent with the pronounced changes in myotube diameter, *Jup* KD inhibited the downregulation of muscle atrophy-related markers (MuRf-1 and Atrogin-1) in *Optn*-overexpressing C2C12 cells (Fig 5C–5G). Together, these data indicate that JUP serves as a novel interacting partner of OPTN in skeletal muscle, and OPTN alleviates Dex-induced muscle atrophy in a JUP-dependent manner.

### PI3K-AKT signaling pathway is down-regulated in *Optn*-KD induced muscle atrophy

**Fig 6:**
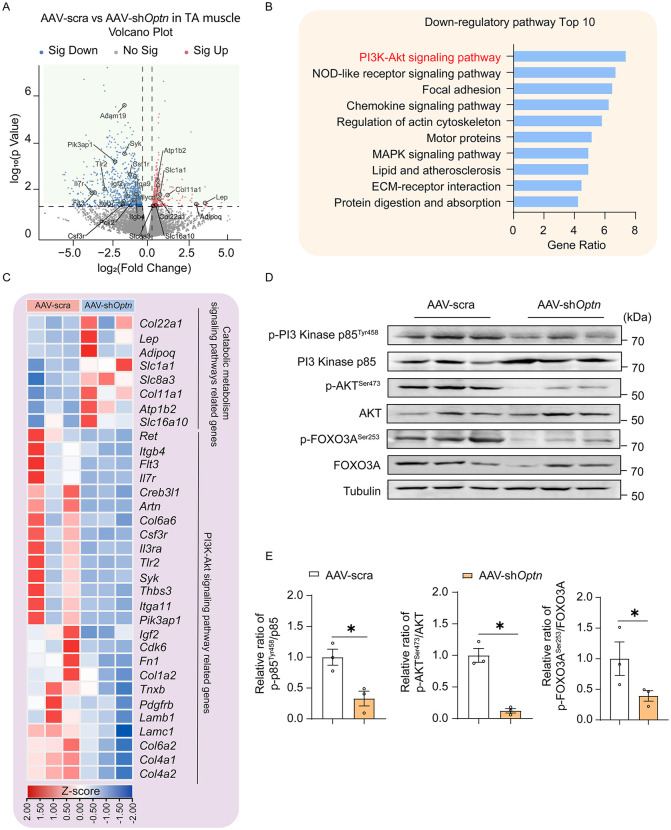
*Optn* knockdown downregulates PI3K-AKT signaling pathway in skeletal muscle. RNA sequencing was performed on TA muscle of *Optn* KD mice and controls. **(A)** Volcano plot of significantly up (red) and downregulated genes (blue). Not significantly changed genes were indicated in gray. The representative genes related PI3K-AKT signaling pathway and catabolic metabolism signaling pathway were labeled on the volcano plot. Red and blue highlighted fold changes of 1.2 and −1.2. *P* value < 0.05. **(B)** KEGG pathway enrichment analysis of significantly changed genes in (A). **(C)** Heatmap showing expression changes in PI3K-AKT and catabolic metabolism signaling pathway-related genes in TA muscle from *Optn* KD and control mice by RNA-seq. **(D, E)** Representative immunoblotting analysis (D) and quantification (E) of PI3K-AKT pathway in scramble shRNA or sh*Optn* TA muscle (*n* = 3 mice in each group). Data are presented as mean ± standard error of the mean (SEM). ^*^P < 0.05 vs. control. The underlying data for this figure can be found in S1 Data. The Original blot for this figure can be found in S1 Raw Image.

To further explore the downstream pathway of OPTN-JUP axis-mediated muscle atrophy, we performed RNA-seq analysis in TA muscle of *Optn* KD and control mice and identified differentially expressed genes (Fig 6A). KEGG analysis showed a high implication of PI3K-AKT pathway (Fig 6B), a characterized pathway in muscle growth and atrophy, with down-regulation of PI3K-AKT pathway-related genes in *Optn* KD mice (Fig 6C). In line with this, we found significantly decreased phosphorylation levels of Tyr458 on PI3 Kinase p85 in TA muscle of *Optn* KD mice (Fig 6D and 6E), which is critical for enhancing PI3K catalytic activity and facilitating downstream signaling [25]. Furthermore, the phosphorylation levels of AKT and FOXO3A were also reduced in TA muscle of *Optn* KD mice (Fig 6D and 6E), indicating that *Optn* KD inhibits the activation of PI3K-AKT pathway in skeletal muscle. These results suggest that PI3K-AKT pathway is the downstream effector of OPTN.

### Pharmacological activation of PI3K-AKT signaling pathway prevents OPTN-KD-induced muscle atrophy

**Fig 7:**
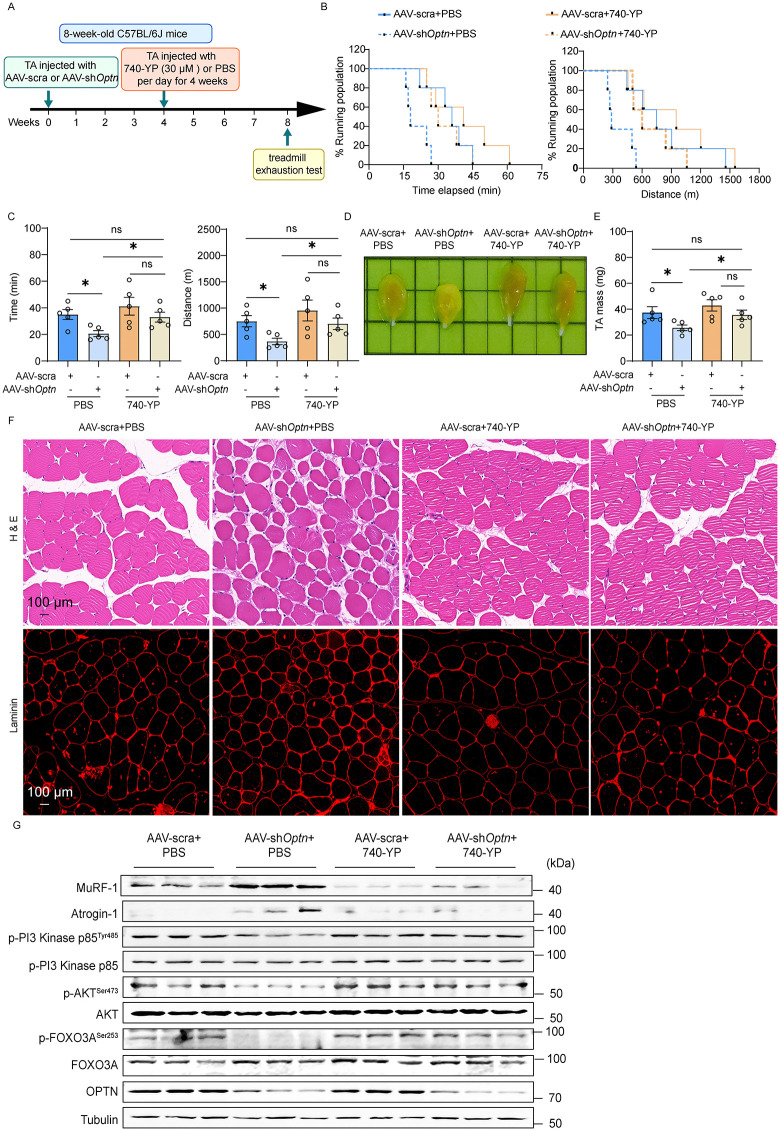
Activation of PI3K-AKT signaling pathway ameliorates *Optn* KD-induced muscle atrophy. **(A)** Schematics for pharmacological activation of PI3K-AKT signaling pathway by 740-YP in *Optn*-KD mice. Four weeks after intramuscular injection of AAV scramble shRNA or AAV-sh*Optn* to TA muscle, mice were then treated with 30 μM 740-YP per day for 4 weeks. **(B, C)** Physical performance was evaluated in mice by a treadmill exhaustion test (*n* = 5 mice in each group). Two parameters were measured with this test: (B) Time (Left panel) and Running distance (Right panel) to exhaustion (Survival plot showing the percentage of mice running at indicated time points and distances). (C) Quantification of mean duration (left panel) and distance of run to exhaustion (Right panel) (*n* = 5 mice in each group). **(D)** Comparison of representative samples of dissected TA muscle in control or *Optn*-KD mice with 740-YP treatment. **(E)** Quantification of TA muscle mass in (D) (*n* = 5 mice in each group). **(F)** Representative H&E and laminin staining of TA muscle in control or *Optn* KD mice with 740-YP treatment (*n* = 5 mice in each group). Scale bar: 100 μm. **(G)** Representative immunoblotting analysis of muscle atrophy markers (Atrogin-1 and Murf-1) and PI3K-AKT pathway in TA muscle from control or *Optn* KD mice with 740-YP treatment (*n* = 3 mice in each group). Data are presented as mean ± standard error of the mean (SEM). ^*^P < 0.05 vs. control. The underlying data for this figure can be found in S1 Data. The Original blot for this figure can be found in S1 Raw Image.

To determine the role of PI3K-AKT signaling pathway in OPTN-KD induced muscle atrophy, we treated *Optn* KD mice with a specific PI3-kinase activator 740-YP (Fig 7A). We observed improved exercise capacity and endurance in *Optn* KD mice receiving 740-YP treatment (Fig 7B and 7C). Consistent with this, we found that the lower weight (Fig 7D and 7E) and smaller fiber size (Figs 7F, S4A, and S4B) of TA muscle shown in *Optn* KD mice were effectively rescued by 740-YP treatment, accompanied with reduced expression levels of Atrogin-1 and MuRF-1 (Figs 7G and S4C). Furthermore, application of 740-YP significantly restored the phosphorylation levels of PI3 Kinase p85, AKT, and FOXO3A in *Optn* KD mice (Figs 7G and S4C). Together, these data indicate that OPTN KD induces muscle atrophy through down-regulation of PI3K-AKT signaling pathway.

### OPTN coordinates the interaction between JUP and PI3 Kinase p85 to activate the PI3K-AKT pathway in skeletal muscle

**Fig 8:**
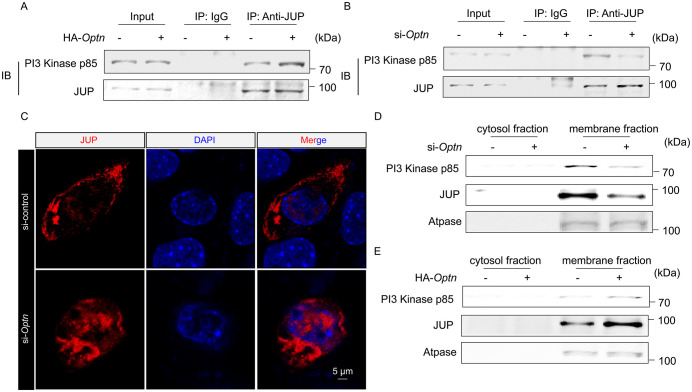
OPTN coordinates the interaction of JUP and PI3 Kinase p85 through targeting JUP to the plasma membrane. **(A, B)** Immunoprecipitation analysis of JUP and PI3 Kinase p85 in *Optn*-overexpressing (A) or KD (B) C2C12 cells. The immunoprecipitation analysis was performed in *Optn*-overexpressing or KD C2C12 cells at 4 d post-differentiation, incubated with anti-JUP antibody or nonspecific Rabbit IgG (control) to pulldown endogenous PI3 Kinase p85 (*n* = 3 biologically independent samples). **(C)** Representative immunofluorescence analysis of JUP in control and *Optn* KD C2C12 cells transfected with Tdtomato-JUP plasmids (*n* = 3 biologically independent samples). Scale bars: 5 μm. **(D, E)** The cytosol and membrane fraction levels of JUP and PI3 Kinase p85 in *Optn* KD (D) or overexpressing (E) C2C12 cells by immunoblotting analysis (*n* = 3 biologically independent samples). The Original blot for this figure can be found in S1 Raw Image.

We further asked how the interaction of OPTN and JUP regulates PI3K-AKT pathway. Given that we and others previously showed that OPTN functions as an autophagy receptor under certain circumstances [10,26], we tested whether OPTN affects the protein levels of JUP. Interestingly, neither *Optn* KD nor overexpression altered protein levels of JUP in TA muscle (S5A and S5B Fig), excluding a possible autophagic degradation of JUP by OPTN. JUP has the capacity to promote PI3K-AKT pathway through binding with PI3 Kinase p85 to plasma membrane [27–29], we thus investigated whether OPTN facilitates JUP-PI3 Kinase p85 interaction. In *Optn*-overexpressing C2C12 cells, JUP interaction with PI3 Kinase p85 was enhanced (Fig 8A), whereas reduced interaction was shown in *Optn* KD cells (Fig 8B). We then examined whether OPTN regulates the plasma membrane levels of PI3 Kinase p85 and JUP. Immunofluorescence staining analysis showed that JUP is localized in the cytoplasm and the cell membrane in myoblasts, while *Optn* KD decreased the proportion of JUP on cell membrane (Fig 8C). Consistent with this, western blot analysis showed decreased membrane fraction of JUP and p85 in *Optn* KD cells (Fig 8D), whereas *Optn* overexpression enhanced JUP and p85 levels in the membrane fraction (Fig 8E).

Finally, we explore the implications of OPTN-JUP interaction in the activation of PI3K-AKT pathway. *Optn*-Δ210-410 overexpression was unable to restore down-regulated phosphorylation levels of PI3 Kinase p85 and AKT in Dex-treated C2C12 cells compared with WT-*Optn* overexpression (S6A and S6B Fig). Consistently, activation of PI3K-AKT signaling pathway in *Optn*-overexpressing cells was abrogated by *Jup* KD (S6C and S6D Fig). Together, these results suggest that OPTN activates PI3K-AKT pathway through coordinating the interaction of JUP and PI3 Kinase p85 in skeletal muscle, and interaction of OPTN and JUP plays a positive role in the activation of PI3K-AKT pathway during muscle atrophy.

## Discussion

Muscle atrophy is a devastating symptom that frequently occurs in sepsis and cachexia, as well as other chronic diseases. Although escalating evidences have been disclosed to unveil the molecular mechanisms of muscle wasting, there are few clinical therapeutic targets for muscle atrophy. Here, we uncovered an active role for OPTN against muscle atrophy. We find that *Optn* knockdown induces muscle atrophy and *Optn* overexpression protects against Dex-induced muscle atrophy by promoting the transduction of PI3K-AKT signaling pathway. Mechanistically, OPTN activates PI3K-AKT signaling pathway through coordinating the interaction of PI3 Kinase p85 and JUP in skeletal muscle. Our finding reveals a new underlying mechanism regulating PI3K-AKT signaling pathway that involves OPTN-JUP complex in maintaining muscle homeostasis, and identify OPTN-mediated protein transportation as a novel therapeutic target for muscle atrophy.

OPTN is a novel therapeutic target for muscle atrophy. Muscle atrophy primarily results from excessive protein breakdown, which is often accompanied by reduced protein synthesis, and leads to a reduced quality of life with increased morbidity and mortality [19]. While growth hormone drugs and myostatin antibodies have shown potential in alleviating muscle atrophy, their safety and effectiveness still need to be further evaluated in clinical trials [30]. Thus, there is an urgent and yet unmet medical need to explore therapeutic targets that will increase muscle mass and strength to improve patient quality of life and survival. OPTN is a protein linked to multiple human degeneration diseases, such as ALS [31]. Our previous study and other researchers’ work showed that OPTN is a new regulator of myogenesis during muscle regeneration or myoblast differentiation [10], suggesting that OPTN is associated with muscle function. However, whether OPTN plays a critical role in muscle atrophy remains unknown. In this study, we found that OPTN protein and mRNA expression are decreased during muscle atrophy in humans and animal models (Fig 1), and *Optn* overexpression in TA muscle protects against Dex-induced muscle atrophy in mice (Fig 3). These results show that OPTN is involved in maintaining muscle mass during muscle atrophy, and identify OPTN as a potential therapeutic target of muscle atrophy. Notably, a recent study reported elevated OPTN expression in denervation-induced muscle atrophy [11]. We propose this discrepancy may stem from distinct pathological mechanisms: denervation triggers compensatory myogenic repair [32], a process known to up-regulate OPTN in our prior work [10], whereas Dex-induced atrophy involves direct catabolic stress without such regenerative activation [19]. In addition, *Optn* KD induces muscle atrophy in mice (Fig 2), including small fiber size and mild pathological morphology with round fibers in TA muscle. These results indicated that OPTN deficiency limits the growth and normal morphology of muscle. Consistently, OPTN suppression causes neuronal cell death and retinal ganglion cell apoptosis [33,34]. Mutation or deletion of human *OPTN* is associated with the risk of cancer (e.g., lung and liver cancer) [35]. These results suggest that OPTN is essential in maintaining cell growth and survival homeostasis; however, the underlying mechanism remains unknown. Therefore, the exact mode of OPTN regulation and the mechanism by which it mediates cell growth and tissue development are important questions in further research.

Muscle atrophy involves the shrinkage of myofibers due to a net loss of proteins, organelles, and cytoplasm, and the autophagy-lysosome pathway is a key degradation system involved in this process [19]. OPTN is a multidomain protein mediating autophagic degradation by interacting with many different proteins, such as LC3 and HECT domain and ankyrin repeat containing E3 ubiquitin protein ligase 1 [26,31]. In present study, we found that OPTN interacts with JUP in skeletal muscle (Fig 6), a positive regulator of PI3K-AKT signaling pathway [18]. A previous study showed that *Jup* KD reduces muscle fiber size through mediating PI3K-AKT signaling pathway in mice [18]. However, *Optn* overexpression and KD do not affect the protein levels of JUP in muscle (S5 Fig). Therefore, OPTN might activate JUP-mediated PI3K-AKT signaling pathway in autophagy-independent manner during muscle atrophy, and multiple functions of OPTN in different tissues and cell types also reveal its greater potential as an efficient target in human diseases.

The PI3K-AKT signaling pathway is a potent anabolic factor that sustains organism and muscle growth [19], and a modulator of insulin receptor activity—JUP was recently identified to regulate the PI3K association with IR on cell membrane during muscle atrophy [18]. Nevertheless, the underlying mechanism of JUP transportation from cytoplasm to cell membrane remains incompletely resolved in muscle [18,29]. It has been reported that OPTN is essential for vesicle trafficking mediated protein transportation in addition to acting as an autophagy receptor [31]. We found that OPTN activates PI3K-AKT signaling pathway via promoting membrane transportation of JUP in muscle atrophy (Fig 8). These findings reveal a novel relationship between OPTN mediated protein transportation and transduction of PI3K-AKT signaling pathway. Similar to our results, OPTN promotes the translocation of EGFR from cytoplasm to membrane through forming a complex with Myosin VI and RAB8a, thereby regulating conduction of the EGFR signaling pathway [36]. Altogether, these results suggest that OPTN-mediated protein transportation plays a key role in the conduction of various intracellular signaling pathways.

In recent years, AAV has been recognized as the leading vehicle (vector) for in vivo delivery of therapeutic genes [37], and the U.S. Food and Drug Administration approvals of AAV-based gene-replacement therapies to treat spinal muscular atrophy and a form of inherited retinal dystrophy highlight the promise of this therapeutic modality [38]. However, there is no application and lack of suitable targets in the clinical treatment of muscle atrophy. In this study, AAV-mediated *Optn* overexpression in TA muscle protects against Dex-induced muscle atrophy and pathological morphology in mice (Fig 3), thus supporting OPTN as a novel target for AAV-based gene therapies of muscle atrophy. However, this study has certain limitations: myofiber-specific OPTN manipulation by skeletal muscle cell-specific promoters is lacking, and AAV delivery was restricted to the TA muscle. Further investigation is warranted to determine the anti-muscle atrophy efficacy of AAV-mediated, cell-type-specific *Optn* overexpression in broader muscle groups (quadriceps muscles, gastrocnemius muscles, etc.) using advanced experimental models, with the goal of maximizing its impact on translational medicine.

**Fig 9:**
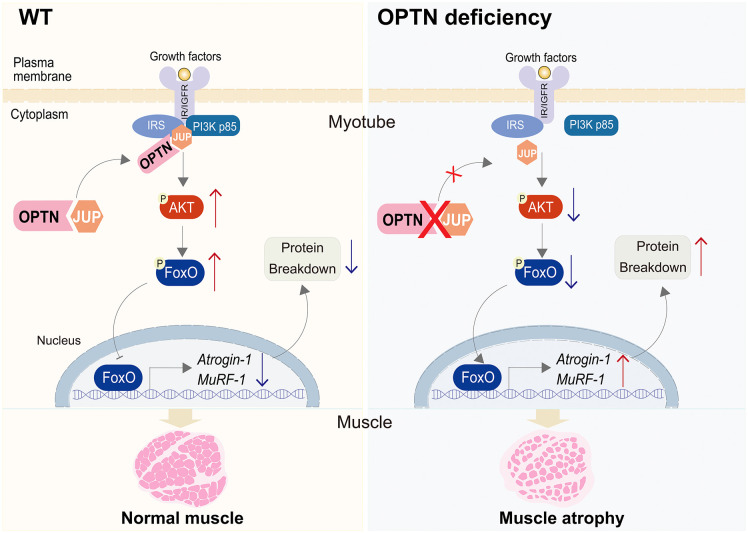
Schematic model illustrating the role of OPTN in muscle atrophy. Left panel. In the presence of OPTN, it binds to JUP and coordinates the interaction between PI3 Kinase p85 and JUP in normal skeletal muscle, promoting activation of the PI3K-AKT pathway. Right panel. OPTN deficiency decreases the binding between PI3 Kinase p85 and JUP, leading to down-regulation of PI3K-AKT pathway. Consequently, the expression levels of Atrogin-1 and Murf-1 were increased, promoting protein breakdown and muscle atrophy. IGFR, insulin-like growth factor receptor; IR, insulin receptor; IRS, insulin receptor substrate; OPTN, optineurin.

In summary, our data identify a new function of OPTN upon muscle atrophy. OPTN deficiency induces muscle atrophy, and overexpression of OPTN prevents muscle atrophy through activating PI3K-AKT signaling pathway. Mechanistically, OPTN promotes the interaction of PI3 Kinase p85 and JUP in skeletal muscle, the key positive regulators of PI3K-AKT signaling pathway (Fig 9). These findings uncover a novel positive role of OPTN through JUP-mediated PI3K-AKT signaling pathway against muscle atrophy, which extends its clinical application and function in human disease.

## Materials and methods

### Animal studies

Six-week-old male C57BL/6J mice purchased from the animal center of Xi’An Jiao Tong University (Xi’an, Shaanxi, PRC) were performed in accordance with the National Institutes of Health (Bethesda, MD, USA) *Guide for the Care and Use of Laboratory Animals* and with the approval of *Animal Ethical and Welfare Committee* of Northwest A&F University (Yang Ling, Shaanxi, PRC) [Approval ID: NWAFU-314031143]. All mice were housed with a 12-h dark/light cycle with food and water ad libitum and were randomly allocated to the indicated groups.

For *Optn* KD in vivo, AAV vectors encoding a control scrambled shRNA sequence (scrambled; 5′-TTCTCCGAACGTGTCACGTAA-3′) or a short hairpin targeting OPTN (sh*Optn*; 5′-GCAAATGGCCATTCTTCTA-3′) under the control of a U6 promoter and expressing EGFP (driven by a CMV promoter) were obtained from Hanbio (Shanghai, PRC). A single dose of 1.1 × 10^12^ vg/mice in 40 μL of AAV2/9 expressing sh*Optn* was delivered to eight-week-old mice injected locally into TA muscle, and the same dose of AAV2/9 expressing shRNA control was injected into TA muscle as AAV-shRNA control group.

For *Optn* OE in vivo, AAV vectors encoding mice *Optn* (HBAAV2/9-CMV-m-OPTN-3xflag-ZsGreen, AAV-m*Optn*) or AAV-Zsgreen (HBAAV2/9-ZsGreen) served as negative control were obtained from Hanbio (Shanghai, PRC). A single dose of 1.8 × 10^12^ vg/mice in 40 μL of AAV2/9 expressing mice *Optn* was delivered to eight-week-old mice injected locally into TA muscle, and the same dose of 1.3 × 10^12^ vg/mice of AAV2/9 expressing Zsgreen was injected into TA muscle as AAV negative control group. Mice were treated after recombinant AAV injection for 4 weeks.

For Dex-induced muscle atrophy, Dex was administered intraperitoneally at 25 mg·kg^-1^ per day for 2 weeks.

To verify the activation of PI3K-AKT signaling pathway in vivo, the TA muscle of scrambled shRNA or sh*Optn* was injected with 20 ul the specific PI3K activator 740-YP (30 μM, HY-P0175, MedChem Express, Shianghai, PRC) or DMSO per mouse for 4 weeks. The TA muscle was then collected for H&E analysis and western blotting.

### Treadmill exhaustion test

To mice were exercised on a treadmill. The system allowed for six individual mice to be exercised simultaneously. Before the exhaustion test, mice were subjected to an acclimation process for 3 consecutive days with the following program: Day 1: static treadmill band 15 min. Day 2: walking on the treadmill for 15 min (10 m/min). Day 3: running for 10 min (15 m/min). Electric stimulus of 1 Hz was employed to force mice to run. Exhaustion test was conducted on two separate days (2 days resting period in-between) with the following program: 10 m/min for 1 min,15 m/min for 5 min, followed by an increase of 1 m/min every minute until a maximum velocity of 25 m/min. Exhaustion was considered after 5 s permanence on the electric grid on a 1 Hz, 0.15 mA, 163 V electric stimulus. Maximum exercise capacity was estimated from each run-to-exhaustion trial using two parameters: the duration of the run (min) and the distance run (m). Values from the two sessions were averaged to provide exercise capacity. Each animal was considered one experimental unit.

### Cell culture

C2C12 cells were purchased from China Infrastructure of Cell Line Resource and were cultured in growth medium comprising high-glucose Dulbecco’s Modified Eagle medium (DMEM) (H30022.01, HyClone, Connecticut, USA) supplemented with 10% fetal bovine serum (FBS) (Z7186FBS-500, ZETA LIFE, California, USA), 1% penicillin/streptomycin. After 48 h, C2C12 cells were cultured in differentiation medium (high-glucose DMEM supplemented with 2% horse serum and 1% penicillin/streptomycin). For the model of Dex-induced muscle atrophy in vitro, C2C12 cells were treated with 50 μM Dex for 24 or 48 h after 4 days in differentiation medium.

### Plasmids and RNA interference

The HA-tagged full-length *Optn* and *Optn* deletion variants were generated by PCR amplification from complementary DNA (cDNA) of mouse or human, followed by cloning into the pcDNA 3.1-HA vector. The Flag-tagged full-length *Jup* is generated by PCR amplification from cDNA of mouse, followed by cloning into the pcDNA 3.1-Flag vector. The PCR primers for cloning are listed in S1 Table. All constructs were confirmed by DNA sequencing. For transient transfection of plasmids into HEK293T and C2C12 cells, Lipo8000 reagent (C0533, Beyotime Biotechnology, Shanghai, PRC) was used according to the manufacturer’s manual.

The si-control, si-*Optn*, and si-*Jup* were synthesized from GenePharma (Shanghai, PRC). The sequences of *Optn* and *Jup* siRNAs were as follows: *Optn* siRNA-GCAAAUGGCCAUUCUUCUATT; *Jup* siRNA-GCUUCAGACUCAAGUACCCATT. For transient transfection of siRNA duplexes into C2C12 cells, Lipo8000 reagent (C0533, Beyotime Biotechnology, Shanghai, PRC) was used according to the manufacturer’s manual.

### Histological analysis

The TA muscle was fixed with 4% paraformaldehyde for more than 72 h and then subjected to dehydration embedding. Finally, paraffin sections of muscle were obtained at a thickness of 2–4 μm for H&E staining, and whole-slide digital images were collected with an Pannoramic DESK Scanner (P-MIDI, P250, 3D HISTECH, Hungary). Cross-sectional area of the myofibers was calculated on section images obtained from TA muscle using Image J.

### Real-time reverse transcriptase PCR

Real-time PCR were performed as described [39]. Total RNA was isolated from the fresh TA muscle using TRIzol reagent (9,109, Takara, Shiga, Japan). Complementary DNA (cDNA) was synthesized from total RNA using cDNA synthesis kit (R333-01, Vazyme Biotech, Nanjing, China) following the manufacturer’s instructions. RT-PCR was performed using a CFX 96 Real-Time PCR Detection System (Bio-Rad, Hercules, CA, USA). Each 20 mL amplification contained 10 μL of ChamQ SYBR qPCR Master Mix (Q222-01, Vazyme Biotech, Nanjing, China), 7.8 mL of sterilized double-distilled water, 1 mL of 1:10 diluted cDNA, and 0.6 mL of each forward and reverse primer. The RT-qPCR program comprised an initial activation step at 95 °C for 3 min, followed by 38 cycles of 95 °C for 15 s, 60 °C for 30 s, and 5 s at 65 °C. After the PCR, a single product generated in these reactions was confirmed via melting curve analysis. The comparative Ct method (2^-ΔΔCt^), described in the literature [40], was used to calculate the gene expression values. The primer sequences for genes were listed in S1 Table.

### Extraction of plasma membrane proteins

The extraction of membrane proteins in C2C12 cells and TA muscle was determined using a plasma membrane protein extraction kit (Invent Biotechnologies, catalogue No. SM-005). The detailed protocol was as follows: C2C12 cells and TA muscle were lysed with buffer A. The filter cartridge was capped and centrifuged at 16,000*g* for 30 s. The filter was discarded, and the pellet was resuspended and centrifuged at 700*g* for 1 min (the pellet contained the intact nuclei). The supernatant was carefully transferred to a new tube and centrifuged for 30 min at 16,000*g*. The supernatant was removed, and the pellet was saved. The total membrane protein fraction was resuspended in buffer B and centrifuged at 7,800*g* for 5 min. The pellet contained the organelle membrane proteins. The supernatant was carefully transferred to a fresh microcentrifuge tube, and mixed a few times by inverting, and centrifuged at 16,000*g* for 30 min. The supernatant was discarded, the pellet (isolated plasma membrane proteins) was saved, and the BCA method was used to determine the protein concentration. Protein samples of membrane fractions were denatured and prepared for immunoblotting.

### Immunoblotting

C2C12 cells and TA muscle were washed with PBS and lysed in RIPA lysis buffer (P0013C, Beyotime Biotechnology, Shanghai, PRC). Next, 200 μg of total protein was resolved by 10% or 12% sodium dodecyl sulfate-polyacrylamide gel (SDS-PAGE) electrophoresis and transferred onto a polyvinylidene fluoride (PVDF) (IPVH00010, Millipore, MA, USA) membrane via electroblotting. The PVDF membrane was blocked in black buffer (5% skim milk powder dissolved in TBST) for 2 h at room temperature. Primary antibodies listed in S2 Table were applied in TBST at 4 °C overnight. Subsequently, the PVDF membrane was washed 4 times with TBST (5 min per time) and stained with the secondary antibodies (goat anti-rabbit or mouse) for 2 h at room temperature. After washing with TBST, the ECL Reagent (WBKlS0100, Millipore, MA, USA) was used, and the strips were on film.

### RNA-sequencing and bioinformatic analyses

The TA muscle samples from *Optn* KD and control mice were used for RNA-seq analyses. Analyses were performed by Majorbio, using the Illumina Noveseq 6000 platform. The RNA-seq data were aligned to corresponding reference genomes (mm10) using HiSat2 and TopHat2. All RNA sequencing data are available in the NCBI Sequence Read Archive under accession code PRJNA1180983.

### Immunoprecipitation

For immunoprecipitation analysis, the TA muscle and cultured cells were homogenized with IP lysis buffer (containing 1M pH 7.4 Tris-HCl 25 mL, NP40 25 mL, NaCl 4.383 g, EDTA 0.146 g, glycerin 50 mL and protease inhibitor cocktail), and the total protein was incubated with Anti-Flag magnetic beads (B26101, Bimake, Shanghai, PRC) and anti-HA magnetic beads (B26201, Bimake, Shanghai, PRC) at 4 °C overnight, or with the antibodies [OPTN (3 µg), JUP (1:100) or nonspecific Rabbit IgG (3 µg)] for 2 h at room temperature followed by the addition of protein A/G magnetic beads (B23201, Bimake, Shanghai, PRC) at 4 °C overnight. After washing three times with TPBS (5 min per time), the protein-bound beads were finally resuspended in 20 μL 1 × SDS-PAGE loading buffer. The samples were boiled at 95 °C for 10 min, and the supernatant was loaded on the gel for immunoblotting.

### IP-MS analysis

OPTN and its interacting protein were immunoprecipitated as described above in the immunoprecipitation assay. Coomassie staining was used to resolve samples run on a 4%–12% SDS-PAGE gel. The whole gel bands were placed in 1.5 mL Eppendorf tubes and then cut into 1 × 1 mm squares. The mass spectrometry sample preparation and purification procedure are followed by the cut gels as previous study [41]. Experiments were carried out using a mass spectrometer in conjunction with liquid chromatography. The RAW data in S2 Data were processed for analysis using Byonic v3.2.0 (Protein Metrics, San Carlos, CA) to identify peptides and infer proteins using the Uniprot Homo sapiens (Human) database (82,492 protein groups). The criteria for selecting the candidate molecules were as follows: 1) the candidates should be presented in the HA-*Optn* overexpressing group but be diminished in the HA-vector overexpressing group, and 2) the number of unique peptides should be > 1.

### Immunofluorescence

Muscle sections and cultured cells were fixed in 4% formaldehyde for 10 min, permeabilized with 0.2% Triton X-100 for 20 min on ice, and then blocked in 3% bovine serum albumin in PBS for 1 h. The samples were blocked in 5% BSA for 2 h at room temperature. Primary antibodies listed in S2 Table were incubated in blocking buffer at 4 °C overnight. Subsequently, the samples were washed with PBS and stained with the appropriate fluorescently labeled secondary antibodies (fluorescein isothiocyanate or rhodamine) for 1 h at room temperature. After washing with PBS, DAPI (C0060, Solarbio, Beijing, PRC) was used to stain nucleus for 3 min. For immunostaining of cultured cells, images were acquired with a confocal laser scanning microscope (TCS SP8; Leica; Wetzler, Germany).

### Statistical analysis

All experiments were at least performed in three independent experiments. Data are presented as mean ± standard error of the mean and were analyzed by two-tailed Student *t* tests for comparisons between two groups or two-way analysis of variance with Duncan post hoc test for multiple comparisons. Statistical significance was defined as ^*^P < 0.05 versus controls. All data were analyzed using PASW Statistics 20 (SPSS, Chicago, IL, USA).

## Supporting information

S1 Fig OPTN is down-regulated in muscle atrophy models in mice. **(A–D)** Relative mRNA expression of *Optn* in TA of different muscle atrophy models (immobilization, Duchenne Muscular Dystrophy, and aging-induced muscle atrophy, as well as Dex-induced muscle atrophy) in mice (*n* = 6 mice in each group). Data are presented as mean ± standard error of the mean (SEM). ^*^P < 0.05 versus control. The underlying data for this figure can be found in S1 Data.
(TIF)

S2 Fig The efficiency of AAV transduction in mouse TA muscle. **(A)** Representative fluorescence image with laminin staining (red) and quantification of EGFP^+^ myofibers (*n* = 5 mice in each group) of TA muscle following AAV-EGFP containing scramble RNA or sh*Optn* transduction after 4 weeks (scale bars: 20 μm). **(B)** Representative fluorescence image with laminin staining (red) and quantification of ZsGreen^+^ myofibers (*n* = 5 mice in each group) of TA muscle following AAV-ZsGreen containing empty vector or mice *Optn* transduction after four weeks (scale bars: 20 μm). Data are presented as mean ± standard error of the mean (SEM). ^*^P < 0.05 versus control. The underlying data for this figure can be found in S1 Data.
(TIF)

S3 Fig The interaction domain of OPTN and JUP is essential for OPTN-mediated muscle atrophy. **(A)** Representative immunofluorescence analysis of myotubes stained with MYHC (green) in C2C12 cells at 4 d post-differentiation with Dex treatment (*n* = 3 biologically independent samples). The C2C12 cells were transfected with HA plasmid, HA-WT *Optn*, or HA-*Optn-*Δ210-410. Scale bar: 50 µm. **(B)** Quantification of fiber diameter in myotubes is described in (A) (*n* = 50 in each group). **(C, D)** Representative immunoblotting analysis (C) and quantification (D) of muscle atrophy markers (Atrogin-1 and Murf-1) in C2C12 cells at 4 d post-differentiation with Dex treatment (*n* = 3 in each group). The C2C12 cells were transfected with HA plasmid, HA-WT *Optn*, or HA-*Optn-*Δ210-410. Data are presented as mean ± standard error of the mean (SEM). ^*^P < 0.05 versus control. The underlying data for this figure can be found in S1 Data. The Original blot for this figure can be found in S1 Raw Image.
(TIF)

S4 Fig Quantification of myofiber size, muscle atrophy markers, and PI3K-AKT pathway protein expression in TA muscle from control or *Optn* KD mice with 740-YP treatment. **(A, B)** Average and distribution of TA muscle myofiber CSAs in control or *Optn* KD mice with 740-YP treatment (*n* = 5 mice in each group). **(C)** Quantification of muscle atrophy markers (Atrogin-1 and Murf-1) and PI3K-AKT pathway in TA muscle from control or *Optn* KD mice with 740-YP treatment (*n* = 3 mice in each group). Data are presented as mean ± standard error of the mean (SEM). * *P* < 0.05 versus control. The underlying data for this figure can be found in S1 Data.
(TIF)

S5 Fig OPTN does not affect the expression of JUP. **(A, B)** Representative immunoblotting analysis (upper panel) and quantification (lower panel) of JUP expression in *Optn* KD (A) or OE (B) C2C12 cells (*n* = 3 in each group). Data are presented as mean ± standard error of the mean (SEM). ^*^P < 0.05 versus control. The underlying data for this figure can be found in S1 Data. The Original blot for this figure can be found in S1 Raw Image.
(TIF)

S6 Fig The implication of OPTN-JUP interaction in activation of PI3K-AKT pathway. **(A, B)** Representative immunoblotting analysis (A) and quantification (B) of PI3K-AKT pathway in C2C12 cells at 4 d post-differentiation with Dex treatment (*n* = 3 in each group). The C2C12 cells were transfected with HA plasmid, HA-WT *Optn*, or HA-*Optn-*Δ210-410. **(C, D)** Representative immunoblotting analysis (C) and quantification (D) of PI3K-AKT pathway in *Optn* OE C2C12 cells with or without *Jup* KD at 4 d post-differentiation with Dex treatment (*n* = 3 in each group). Data are presented as mean ± standard error of the mean (SEM). ^*^P < 0.05 versus control. The underlying data for this figure can be found in S1 Data. The Original blot for this figure can be found in S1 Raw Image.
(TIF)

S1 Table Primers used in this study. (DOCX)

S2 Table Primary antibodies used in this study. (DOCX)

S1 Data Contains underlying data for Figs 1A; 1C; 2B, 2C; 2E; 2G–2I; 2K; 3B, 3C; 3E; 3G–3I; 3K; 5B; 5D–5G; 6A–6C; 6E; 7B, 7C; 7E; S1A–S1D; S2A, S2B; S3B; S3D; S4A–S4C; S5A, S5B; S6B; S6D. (XLSX)

S2 Data The raw mass spectrometry data file. (XLSX)

S1 Raw Image Original blot contains Figs 1B; 2J; 3J; 4C–4E; 4G; 5C; 6D; 7G; 8A, 8B; 8D, 8E; S3C; S5A, S5B; S6A; S6C. (PDF)

## Abbreviations

ALS: amyotrophic lateral sclerosis
cDNA: complementary DNA
Dex: dexamethasone
DMEM: Dulbecco’s Modified Eagle medium
FBS: fetal bovine serum
GC: glucocorticoid
IP-MS: immunoprecipitation and mass spectrometry analysis
JUP: Junction plakoglobin
KD: Knockdown
MuRF-1: muscle RING finger-1 protein
OPTN: Optineurin
PVDF: polyvinylidene fluoride
SDS-PAGE: sodium dodecyl sulfate polyacrylamide gel
sh*Optn*: short hairpin *Optn*
shRNA: short hairpin RNA
TA: tibialis anterior
