# Supplementary figures and images for: Optineurin binding to the novel interacting partner Junction plakoglobin prevents muscle atrophy in mice
Source: PLoS Biol. 2026 Jan 22;24(1):e3003581. doi: 10.1371/journal.pbio.3003581 (PMC12851441; doi:10.1371/journal.pbio.3003581)

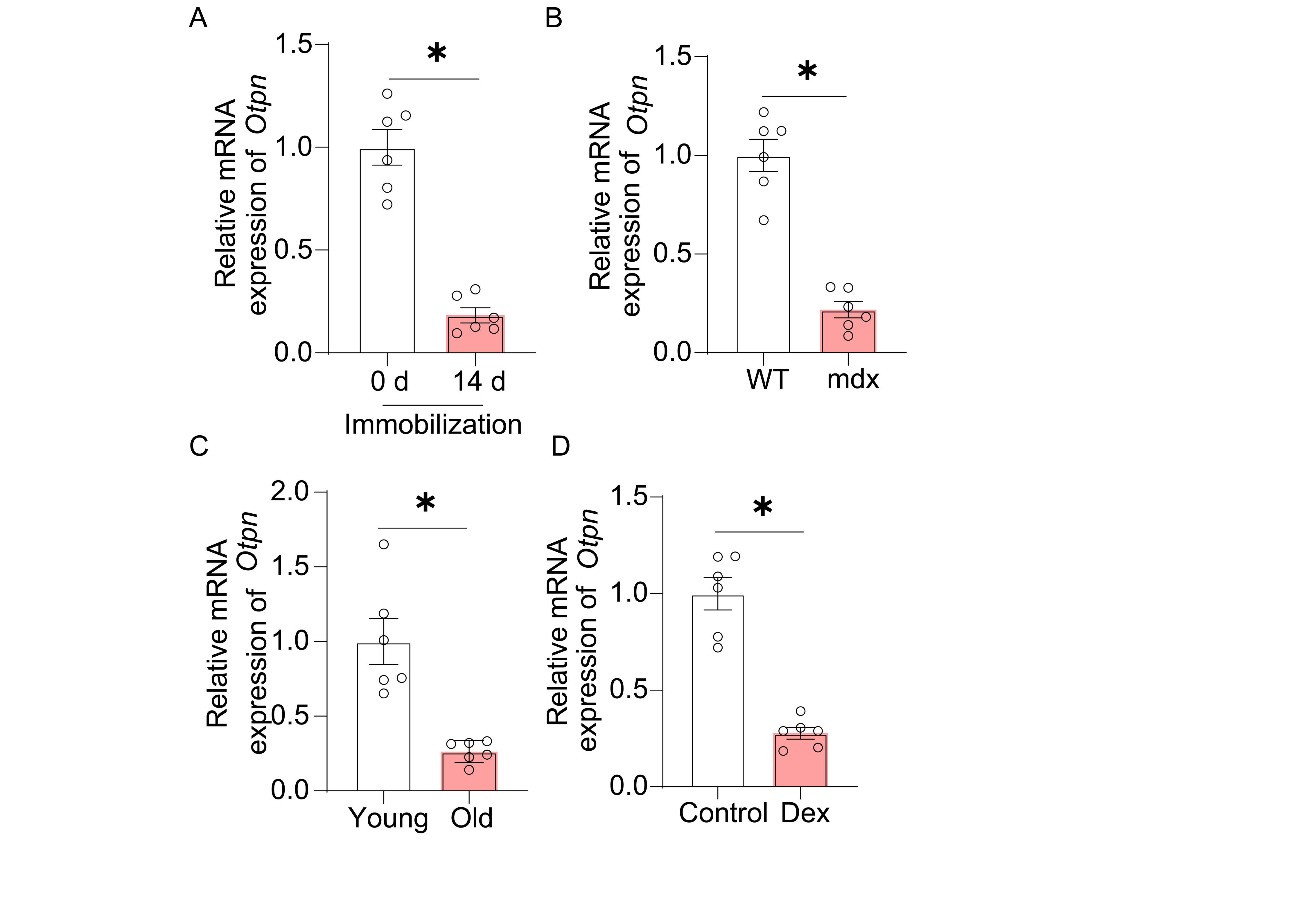

Supplement: S1 Fig — (A–D) Relative mRNA expression of Optn in TA of different muscle atrophy models (immobilization, Duchenne Muscular Dystrophy, and aging-induced muscle atrophy, as well as Dex-induced muscle atrophy) in mice (n = 6 mice in each group). Data are presented as mean ± standard error of the mean (SEM). *P < 0.05 versus control. The underlying data for this figure can be found in S1 Data. (TIF) [file pbio.3003581.s001.tif]

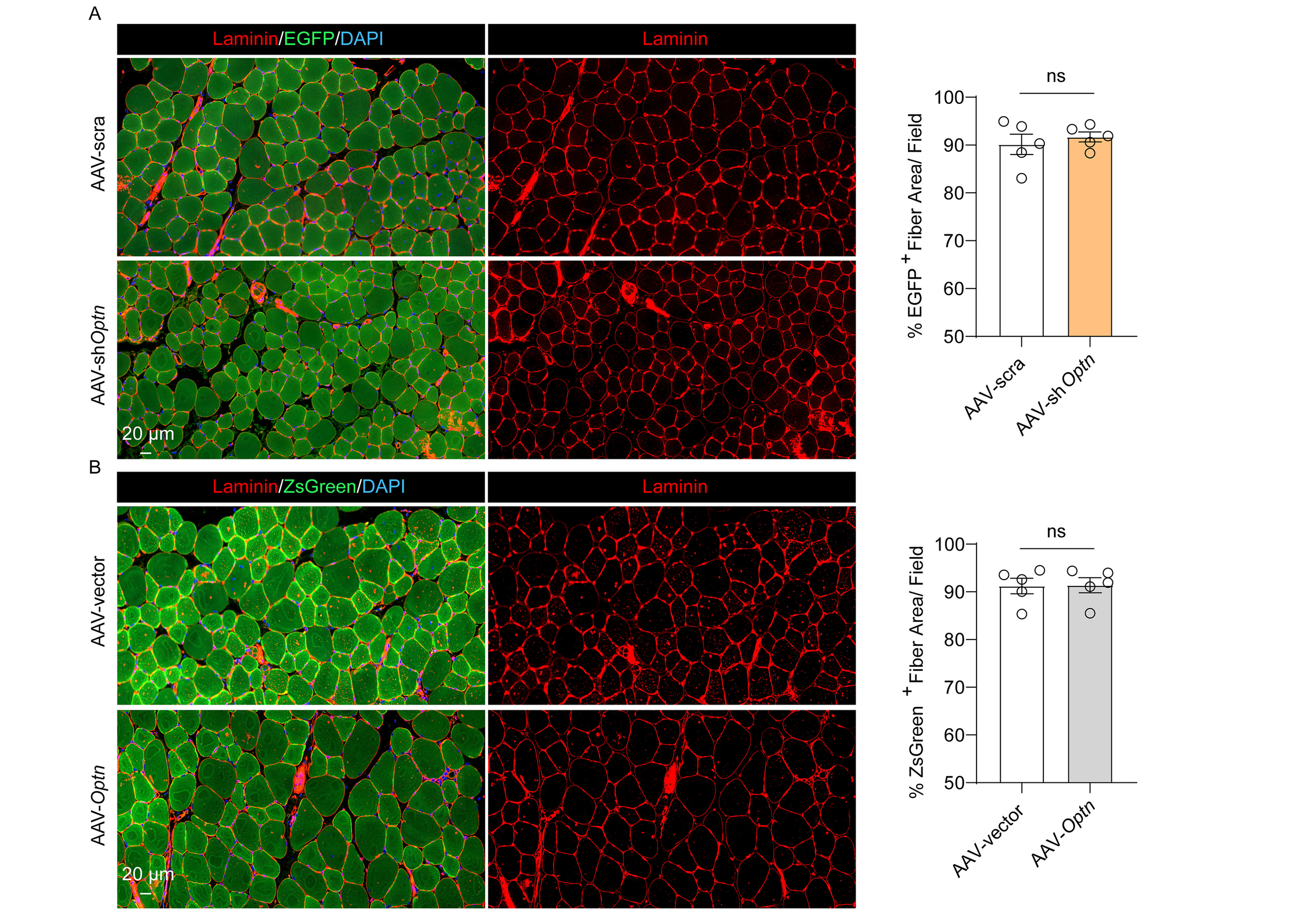

Supplement: S2 Fig — (A) Representative fluorescence image with laminin staining (red) and quantification of EGFP+ myofibers (n = 5 mice in each group) of TA muscle following AAV-EGFP containing scramble RNA or shOptn transduction after 4 weeks (scale bars: 20 μm). (B) Representative fluorescence image with laminin staining (red) and quantification of ZsGreen+ myofibers (n = 5 mice in each group) of TA muscle following AAV-ZsGreen containing empty vector or mice Optn transduction after four weeks (scale bars: 20 μm). Data are presented as mean ± standard error of the mean (SEM). *P < 0.05 versus control. The underlying data for this figure can be found in S1 Data. (TIF) [file pbio.3003581.s002.tif]

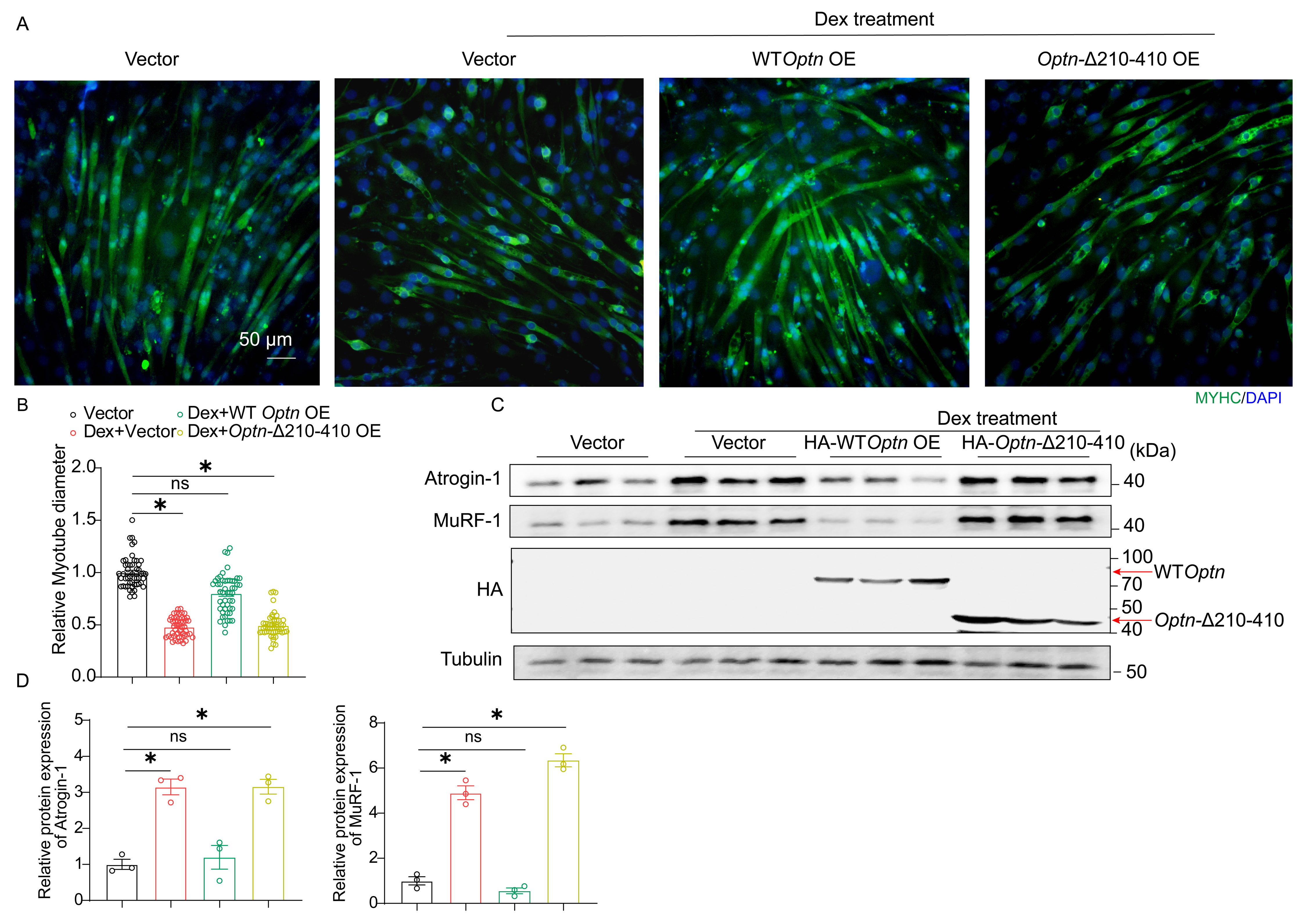

Supplement: S3 Fig — (A) Representative immunofluorescence analysis of myotubes stained with MYHC (green) in C2C12 cells at 4 d post-differentiation with Dex treatment (n = 3 biologically independent samples). The C2C12 cells were transfected with HA plasmid, HA-WT Optn, or HA-Optn-Δ210-410. Scale bar: 50 µm. (B) Quantification of fiber diameter in myotubes is described in (A) (n = 50 in each group). (C, D) Representative immunoblotting analysis (C) and quantification (D) of muscle atrophy markers (Atrogin-1 and Murf-1) in C2C12 cells at 4 d post-differentiation with Dex treatment (n = 3 in each group). The C2C12 cells were transfected with HA plasmid, HA-WT Optn, or HA-Optn-Δ210-410. Data are presented as mean ± standard error of the mean (SEM). *P < 0.05 versus control. The underlying data for this figure can be found in S1 Data. The Original blot for this figure can be found in S1 Raw Image. (TIF) [file pbio.3003581.s003.tif]

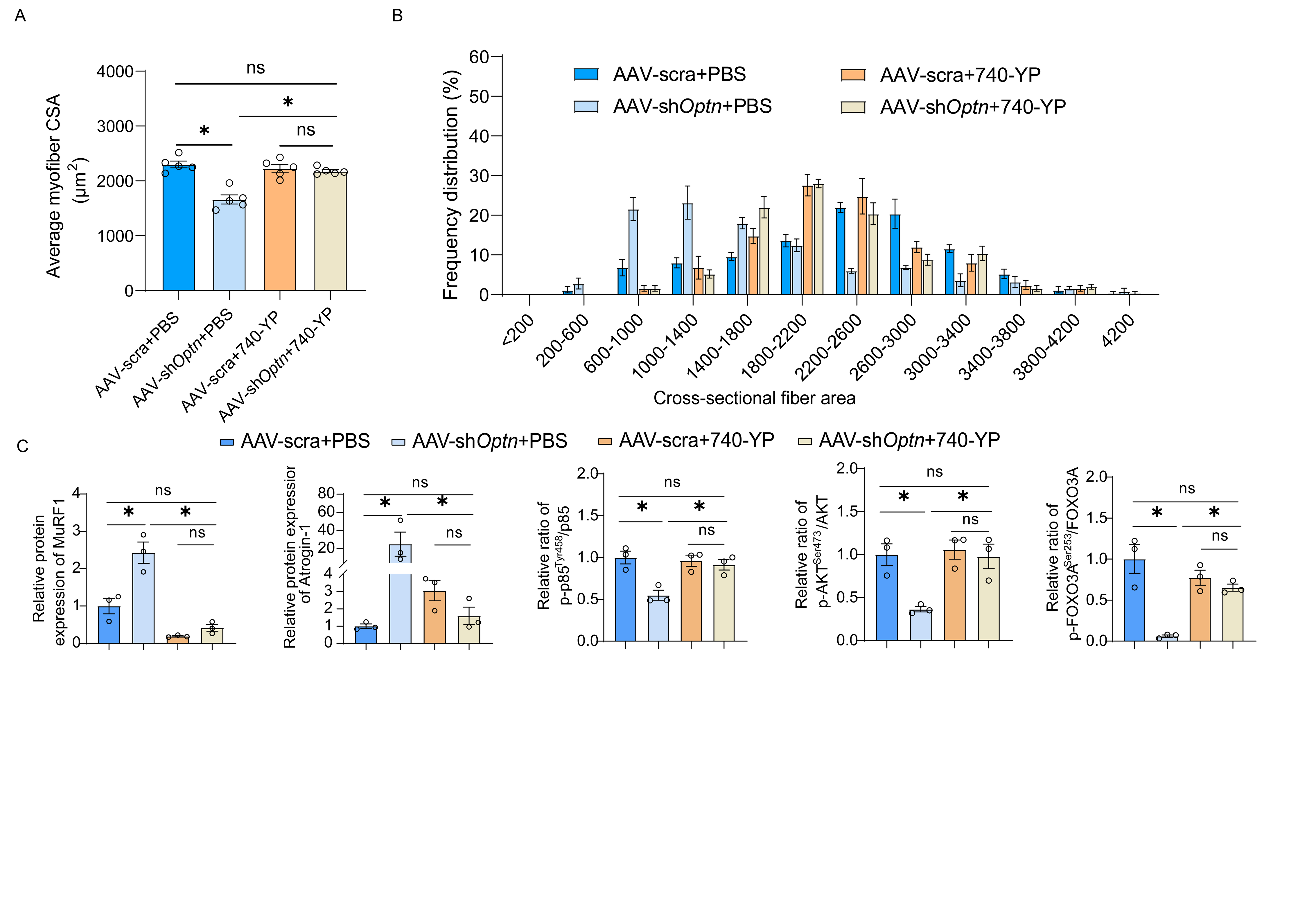

Supplement: S4 Fig — (A, B) Average and distribution of TA muscle myofiber CSAs in control or Optn KD mice with 740-YP treatment (n = 5 mice in each group). (C) Quantification of muscle atrophy markers (Atrogin-1 and Murf-1) and PI3K-AKT pathway in TA muscle from control or Optn KD mice with 740-YP treatment (n = 3 mice in each group). Data are presented as mean ± standard error of the mean (SEM). * P < 0.05 versus control. The underlying data for this figure can be found in S1 Data. (TIF) [file pbio.3003581.s004.tif]

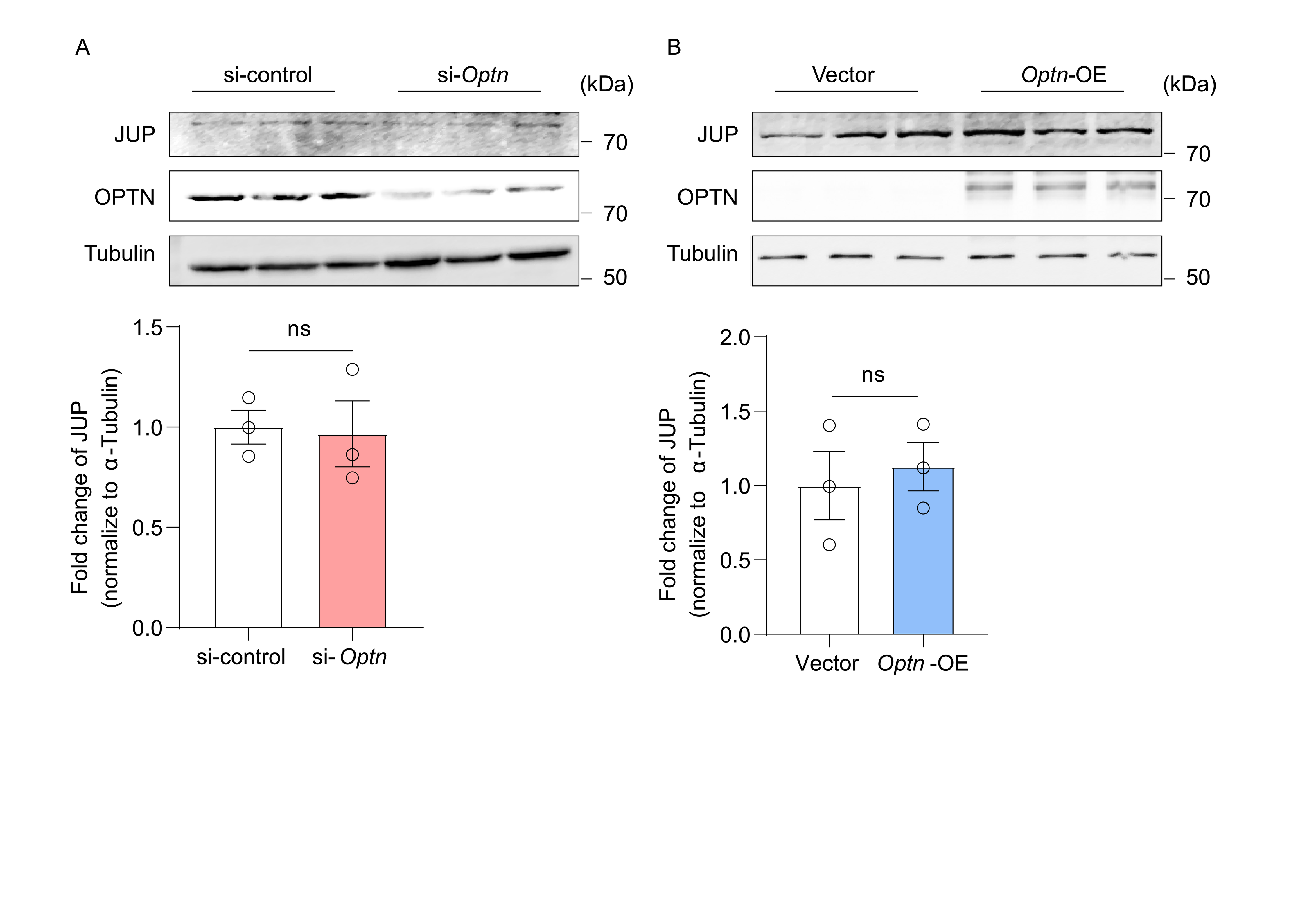

Supplement: S5 Fig — (A, B) Representative immunoblotting analysis (upper panel) and quantification (lower panel) of JUP expression in Optn KD (A) or OE (B) C2C12 cells (n = 3 in each group). Data are presented as mean ± standard error of the mean (SEM). *P < 0.05 versus control. The underlying data for this figure can be found in S1 Data. The Original blot for this figure can be found in S1 Raw Image. (TIF) [file pbio.3003581.s005.tif]

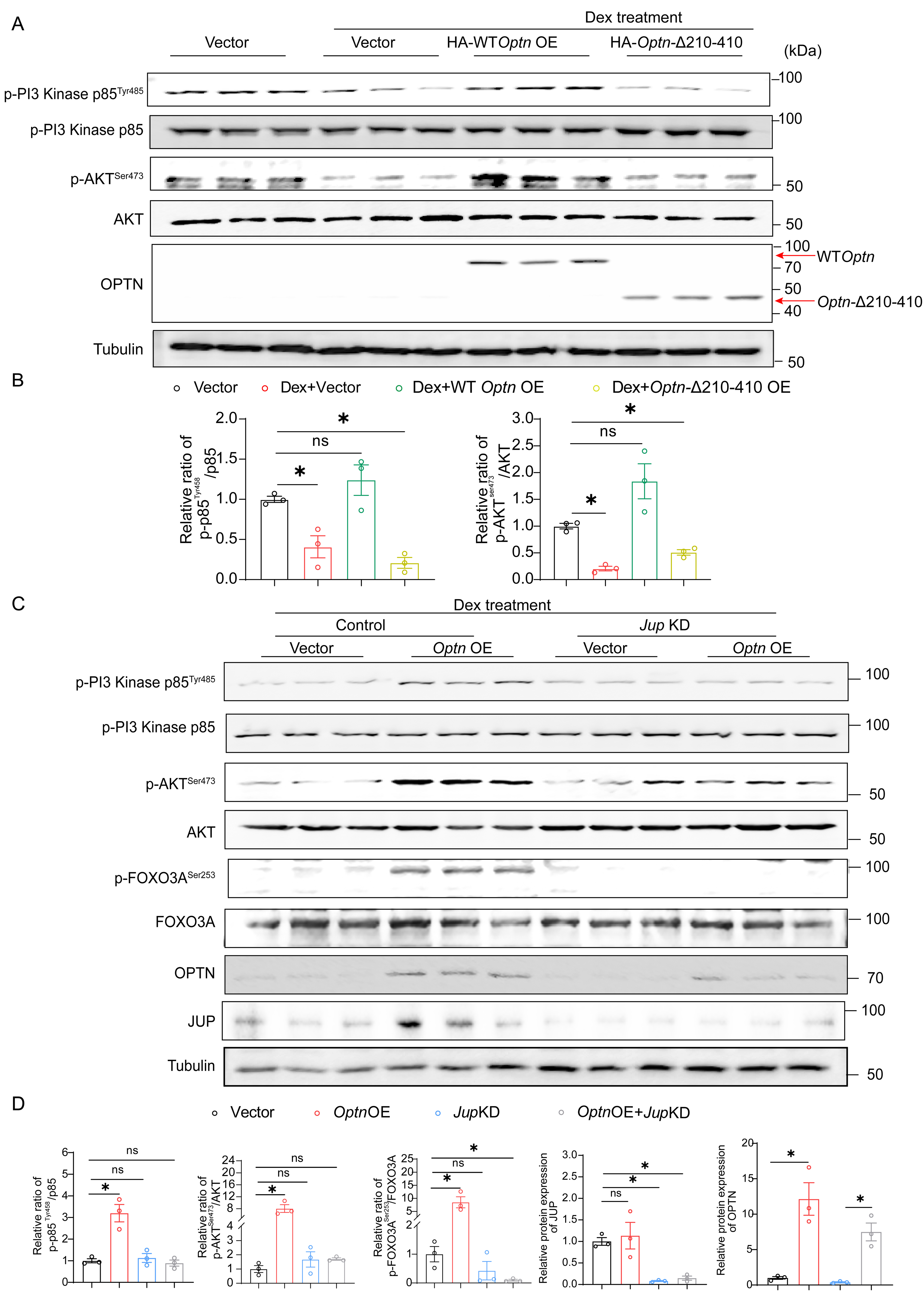

Supplement: S6 Fig — (A, B) Representative immunoblotting analysis (A) and quantification (B) of PI3K-AKT pathway in C2C12 cells at 4 d post-differentiation with Dex treatment (n = 3 in each group). The C2C12 cells were transfected with HA plasmid, HA-WT Optn, or HA-Optn-Δ210-410. (C, D) Representative immunoblotting analysis (C) and quantification (D) of PI3K-AKT pathway in Optn OE C2C12 cells with or without Jup KD at 4 d post-differentiation with Dex treatment (n = 3 in each group). Data are presented as mean ± standard error of the mean (SEM). *P < 0.05 versus control. The underlying data for this figure can be found in S1 Data. The Original blot for this figure can be found in S1 Raw Image. (TIF) [file pbio.3003581.s006.tif]
