# Supplementary material for: Optineurin binding to the novel interacting partner Junction plakoglobin prevents muscle atrophy in mice
Source: PLoS Biol. 2026 Jan 22;24(1):e3003581. doi: 10.1371/journal.pbio.3003581 (PMC12851441; doi:10.1371/journal.pbio.3003581)
Supplement: S1 Table — (DOCX) [file pbio.3003581.s007.docx]

**S1 Table** Primers used in this study.

| Gene | Forward | Reverse |
| --- | --- | --- |
| Primers for qPCR analysis used | | |
| *Optn* | TGTCAGGCTCTGGAGAGGAA | GTCTTGGCCTGCTCCATCTT |
| *Atrogin-1* | AGCGCTTCTTGGATGAGAAA | GGCAGTCGAGAAGTCCAGTC |
| *MuRF-1* | TGCCTGGAGATGTTTACCAAGC | CCATGAGCGCATCGCAATC |
| Primers for plasmid construction used | | |
| HA-m*Optn* | CGCGGATCCATGTCCCATCAACCTCTG | CCGCTCGAGTCAAATGATGCAGTCCATCAC |
| EGFP- m*Optn* | CGCGGATCCATGTCCCATCAACCTCTG | CCGCTCGAGTCAAATGATGCAGTCCATCAC |
| FLAG-m*Jup* | CGGAATTCGCGCCACCATGGAGGTGATGAACCTTATTG | GCTCTAGACTAGGCCAGCATGTGGTCTGCAGTG |
| Tdtomato- m*Jup* | GGAATCCCGGGAGGAGGGGGATCCATGGAGGTGATGAACCTTATTG | GCTCTAGACTAGGCCAGCATGTGGTCTGCAGTG |
| HA-m*Optn* (1-209) | CGCGGATCCATGTCCCATCAACCTCTG | CCGCTCGAGAGGGCAGTTCTTCATCTCCTTCATTGC |
| HA-m*Optn* (210-410) | CGCGGATCCACACCCACAAGAACAGACC | CCGCTCGAGAATTGTTTTCAGTGCCTTATTATGTTC |
| HA-m*Optn* (410-585) | CGCGGATCCGAAGAACTAACCAAGCAACAGGCAG | CCGCTCGAGTCAAATGATGCAGTCCATCAC |
| HA-m*Optn*-Δ210-410 (deletion)  overlap | CGCGGATCCATGTCCCATCAACCTCTG | GTTGCTTGGTTAGTTCTTCAGGGCAGTTCTTCATCTCC |
|  | AAGGAGATGAAGAACTGCCCTGAAGAACTAACCAAGCA | CCGCTCGAGTCAAATGATGCAGTCCATCAC |
