# Supplementary material for: Optineurin binding to the novel interacting partner Junction plakoglobin prevents muscle atrophy in mice
Source: PLoS Biol. 2026 Jan 22;24(1):e3003581. doi: 10.1371/journal.pbio.3003581 (PMC12851441; doi:10.1371/journal.pbio.3003581)
Supplement: S2 Table — (DOCX) [file pbio.3003581.s008.docx]

**S2 Table** Primary antibodies used in this study.

| Antibody | Dilution (WB/IF/IP) | Source | Cat. No |
| --- | --- | --- | --- |
| OPTN | 1:1000/no/3 ug | Proteintech | 10837-1-AP |
| JUP | 1:1000/1:400/1:100 | Cell Sinaling Technology | 2309 |
| Atrogin-1 | 1:1000/no/no | Santa Cruz | sc-166806 |
| MuRF-1 | 1:1000/no/no | Santa Cruz | sc-398608 |
| p-FOXO3A^ser253^ | 1:1000/no/no | Immunoway | YP0115 |
| FOXO3A | 1:1000/no/no | Immunoway | YT1763 |
| p-AKT^Ser473^ | 1:2000/no/no | Cell Sinaling Technology | 4060 |
| AKT | 1:1000/no/no | Cell Sinaling Technology | 9272 |
| p-PI3 Kinase p85^Tyr458^ | 1:1000/no/no | Cell Sinaling Technology | 4228 |
| PI3 Kinase p85 | 1:1000/no/1:50 | Cell Sinaling Technology | 4257 |
| p-mTOR^ser2448^ | 1:1000/no/no | Cell Sinaling Technology | 5536 |
| mTOR | 1:1000/no/no | Cell Sinaling Technology | 2983 |
| α-TUBULIN | 1:4000/no/no | Proteintech | 11224-1-AP |
| Atpase | 1:20000/no/no | Proteintech | 14418-1-AP |
| Laminin | no/1:100/no | Abcam | ab11575 |
| MYHC | 1:2000/1:100/no | R&D systems | MAB4470 |
| Goat anti-rabbit (HRP) | 1:10000/no/no | Abbkine | A21020 |
| Goat anti-mouse (HRP) | 1:10000/no/no | Abbkine | A21021 |
