# Supplementary material for: Optineurin binding to the novel interacting partner Junction plakoglobin prevents muscle atrophy in mice
Source: PLoS Biol. 2026 Jan 22;24(1):e3003581. doi: 10.1371/journal.pbio.3003581 (PMC12851441; doi:10.1371/journal.pbio.3003581)

Figure 1

Fig 1B.

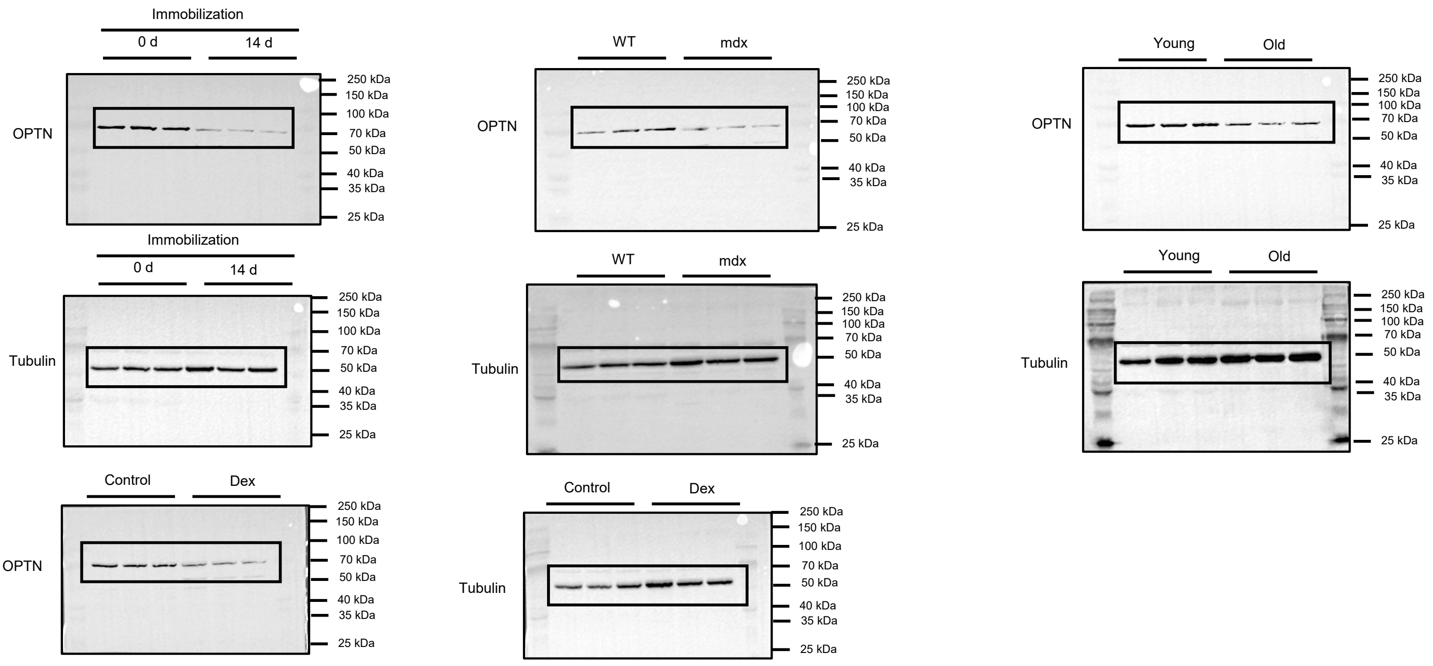

Figure 2

Fig 2J.

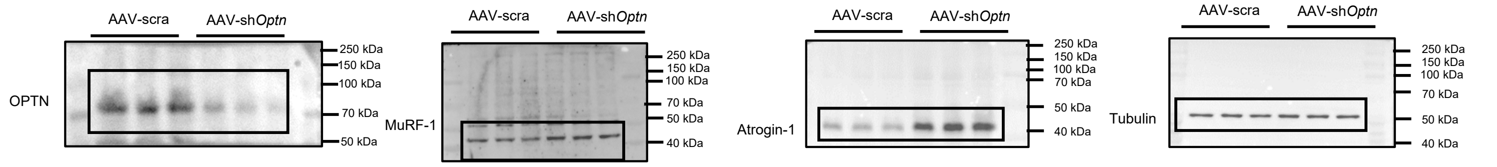

Figure 3

Fig 3J.

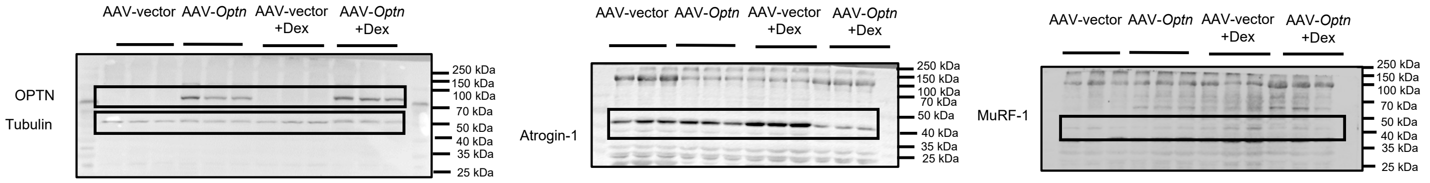

Figure 4

Fig 4C.

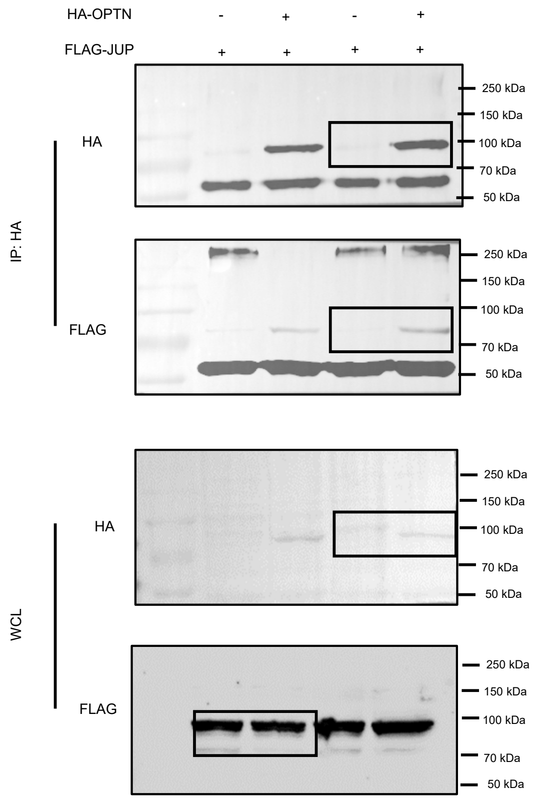

Fig 4D.

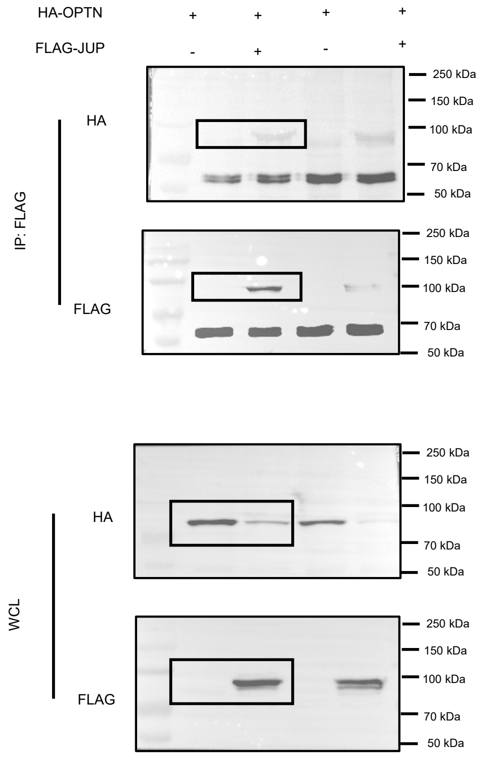

Fig 4E.

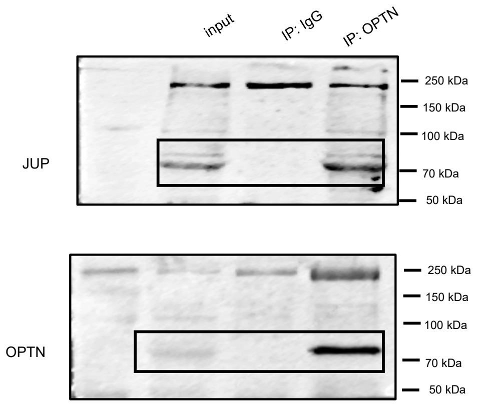

Fig 4G.

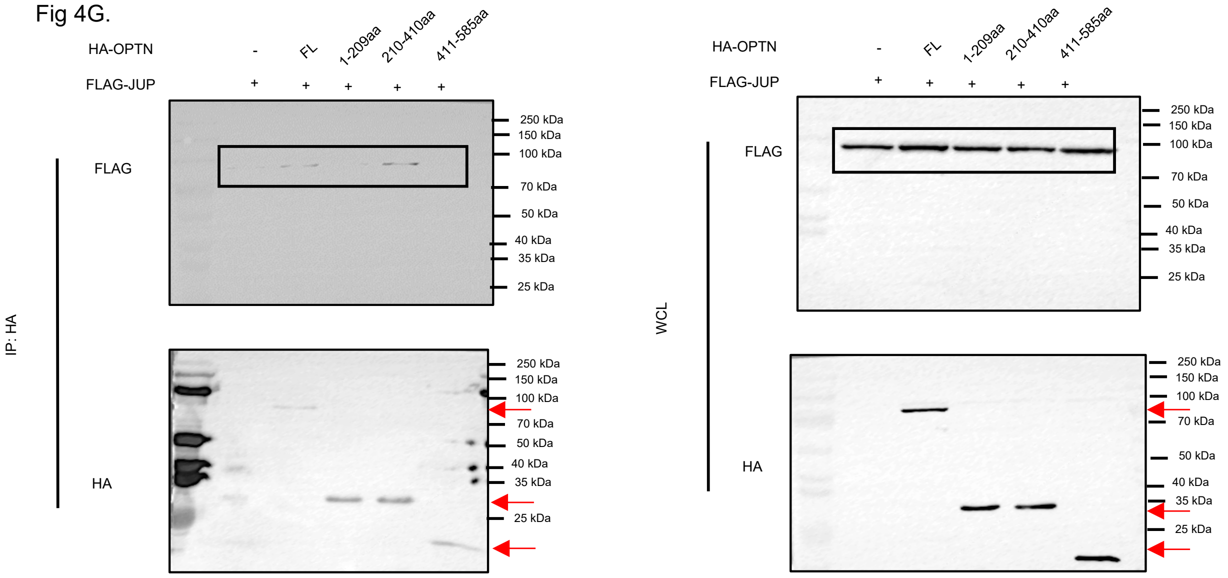

Figure 5

Fig 5C.

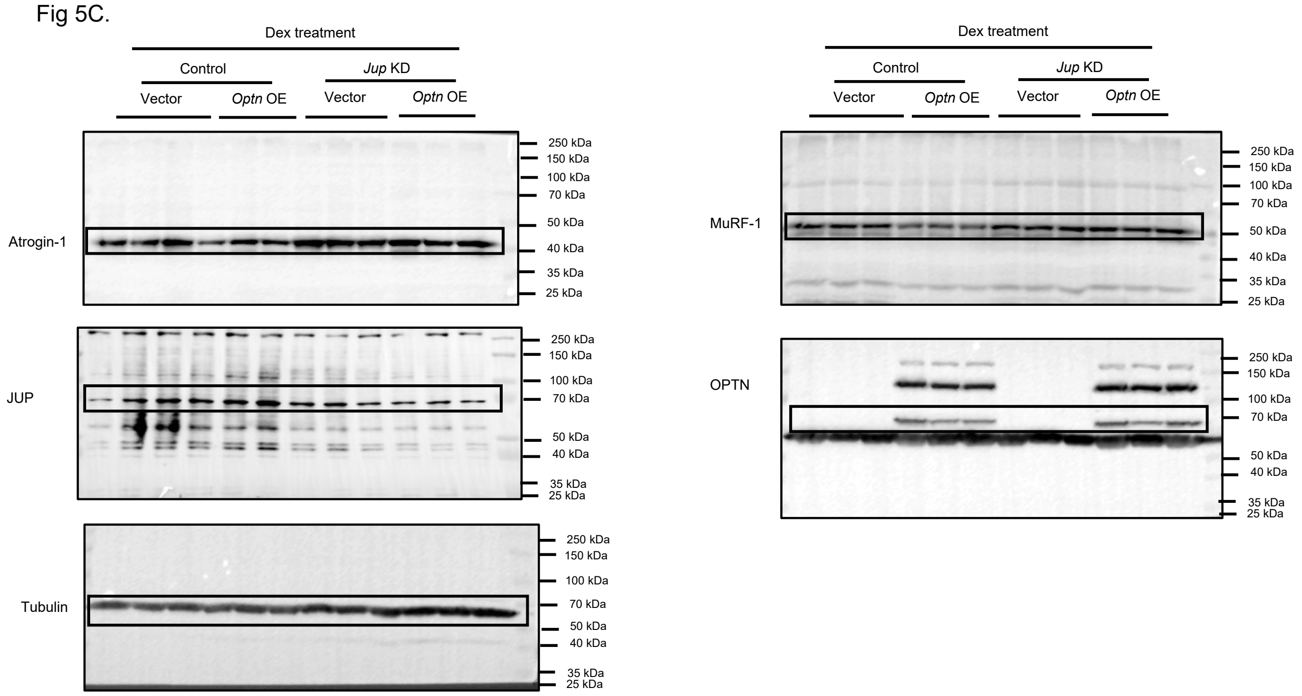

Figure 6

Fig 6D.

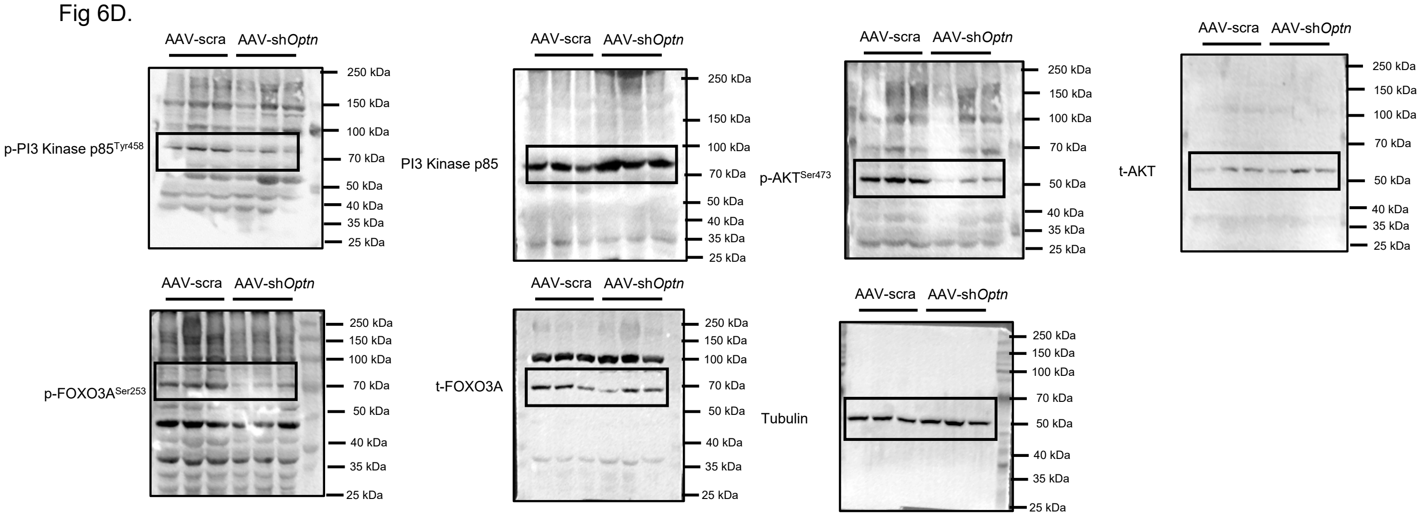

Figure 7

Fig 7G.

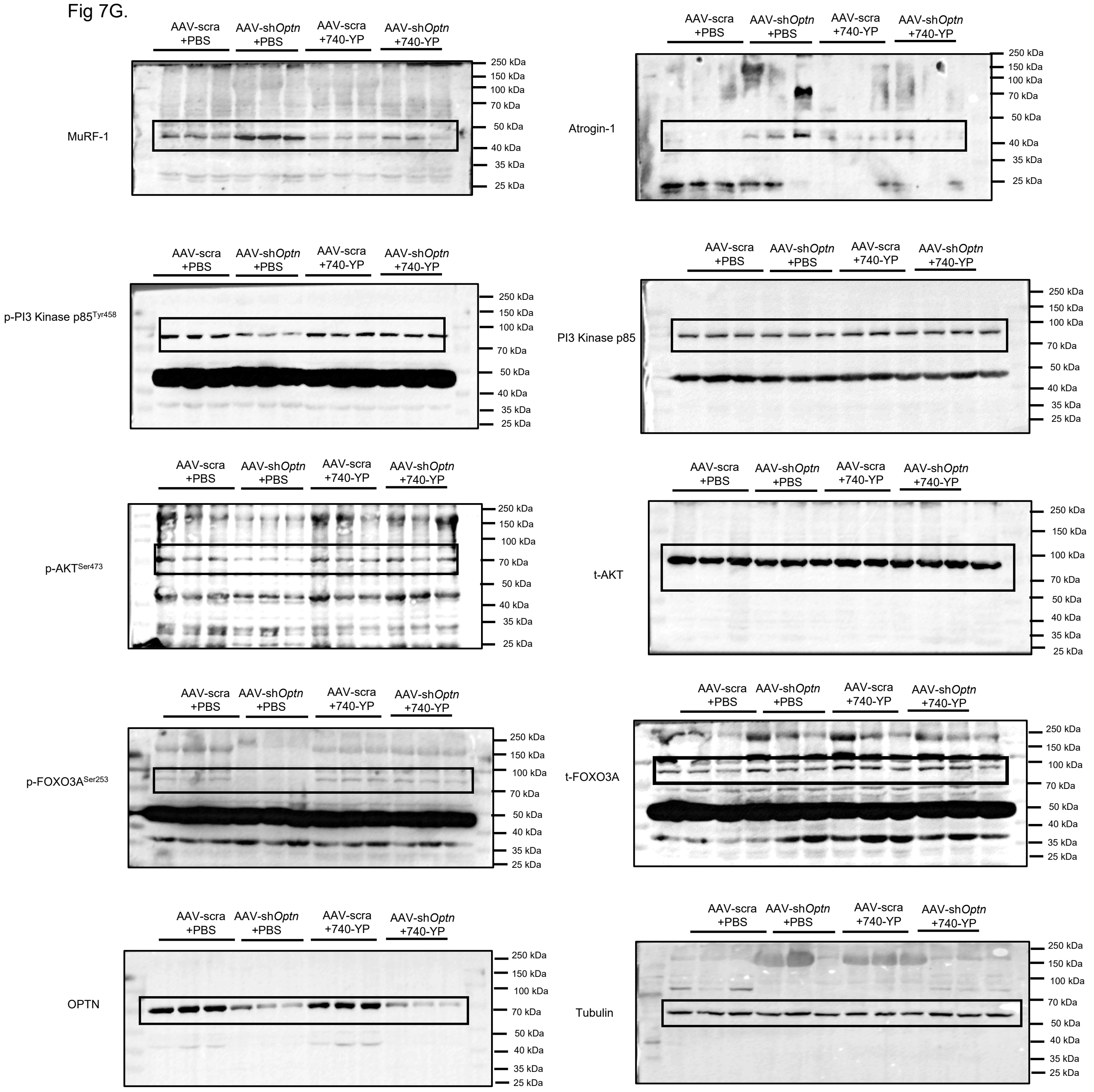

Figure 8

Fig. 8A

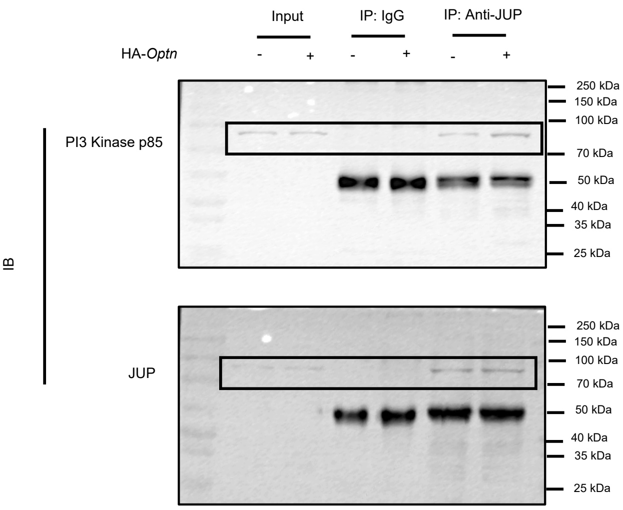

Fig. 8B

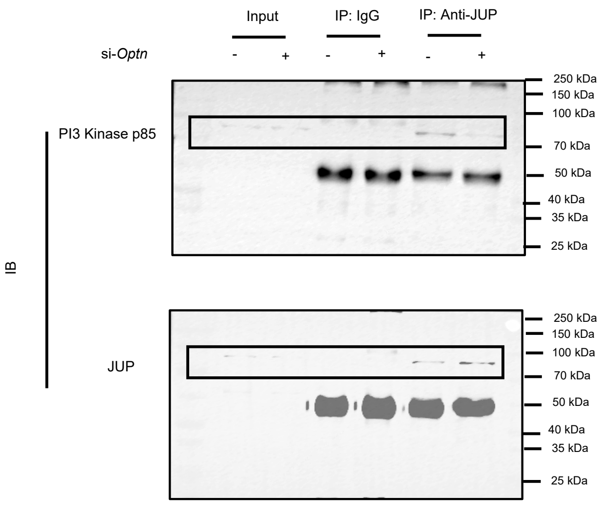

Fig. 8D

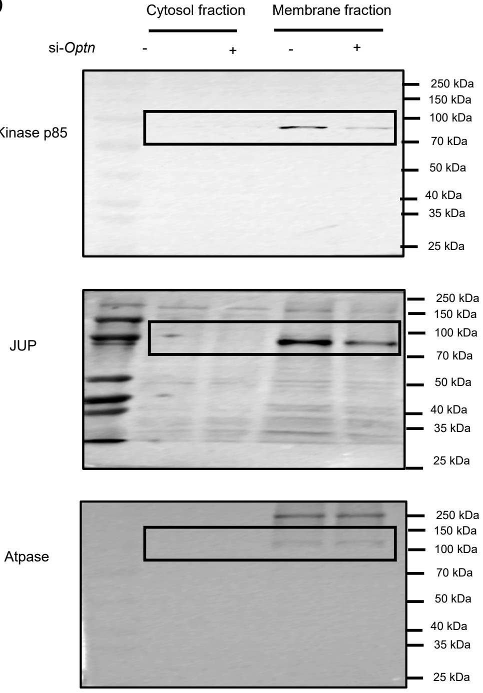

Fig. 8E

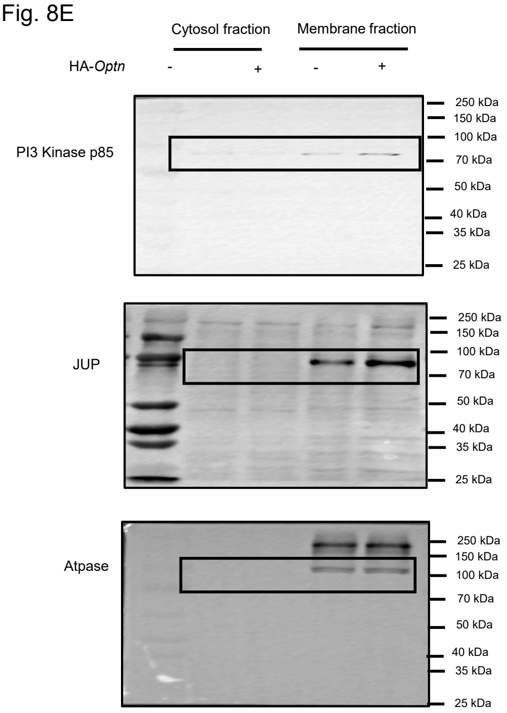

S3 Figure

S3C Fig.

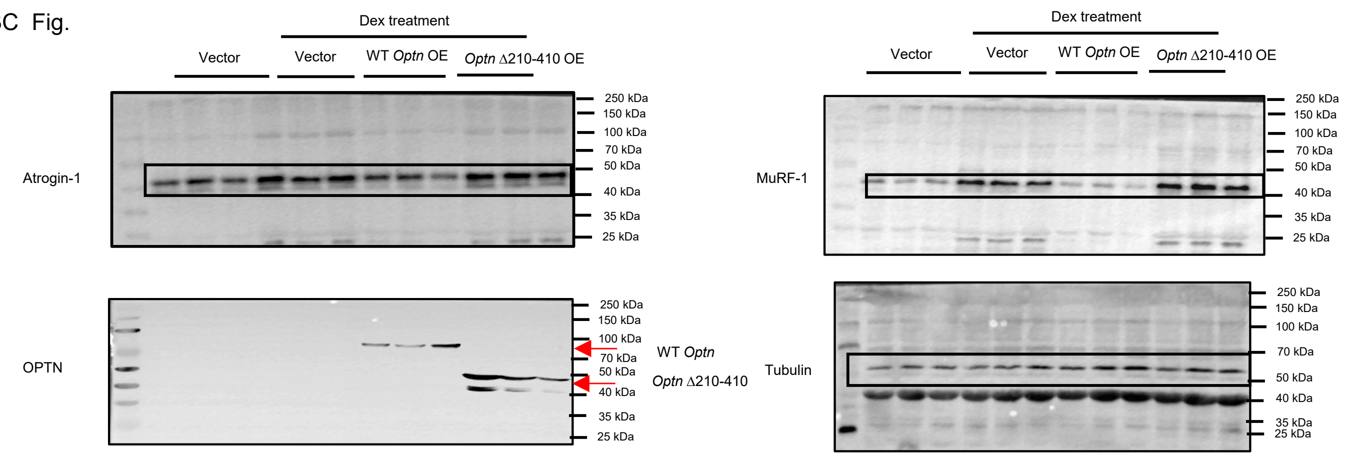

S5 Figure

S5A Fig.

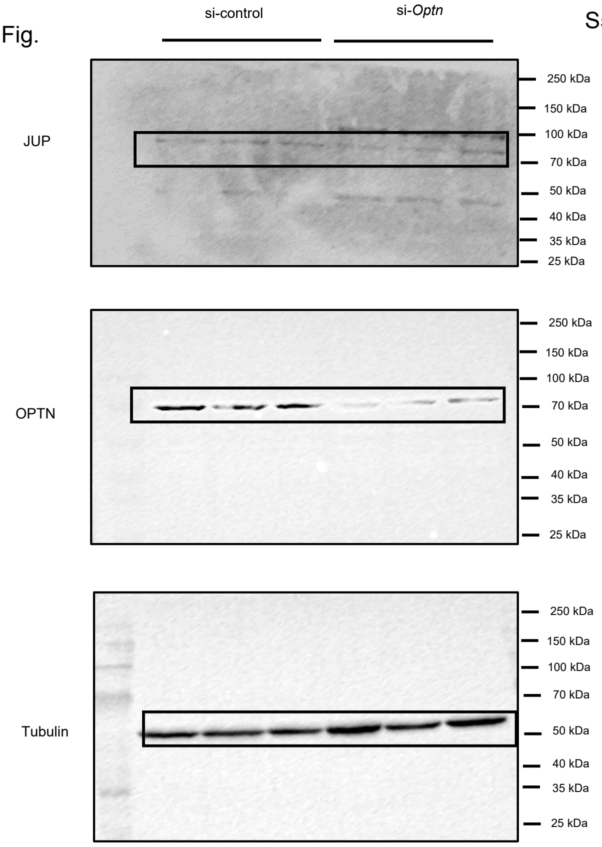

S5B Fig.

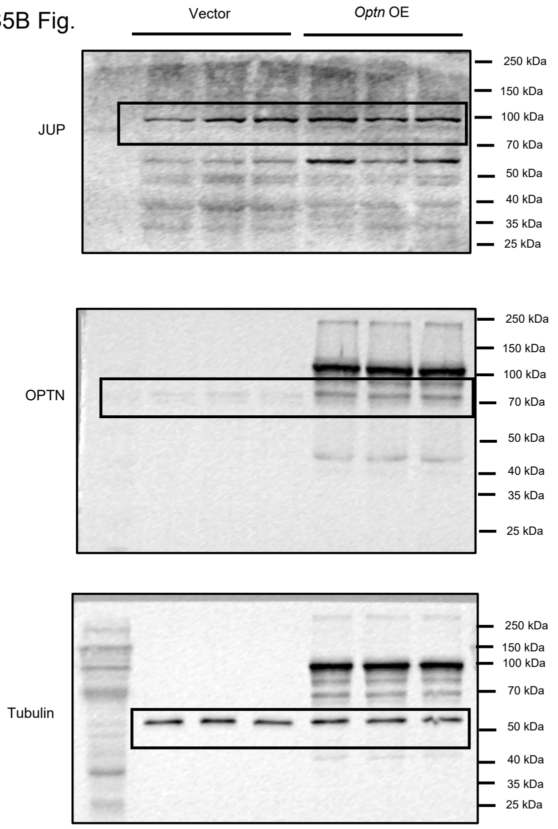

S6 Figure

S6A Fig.

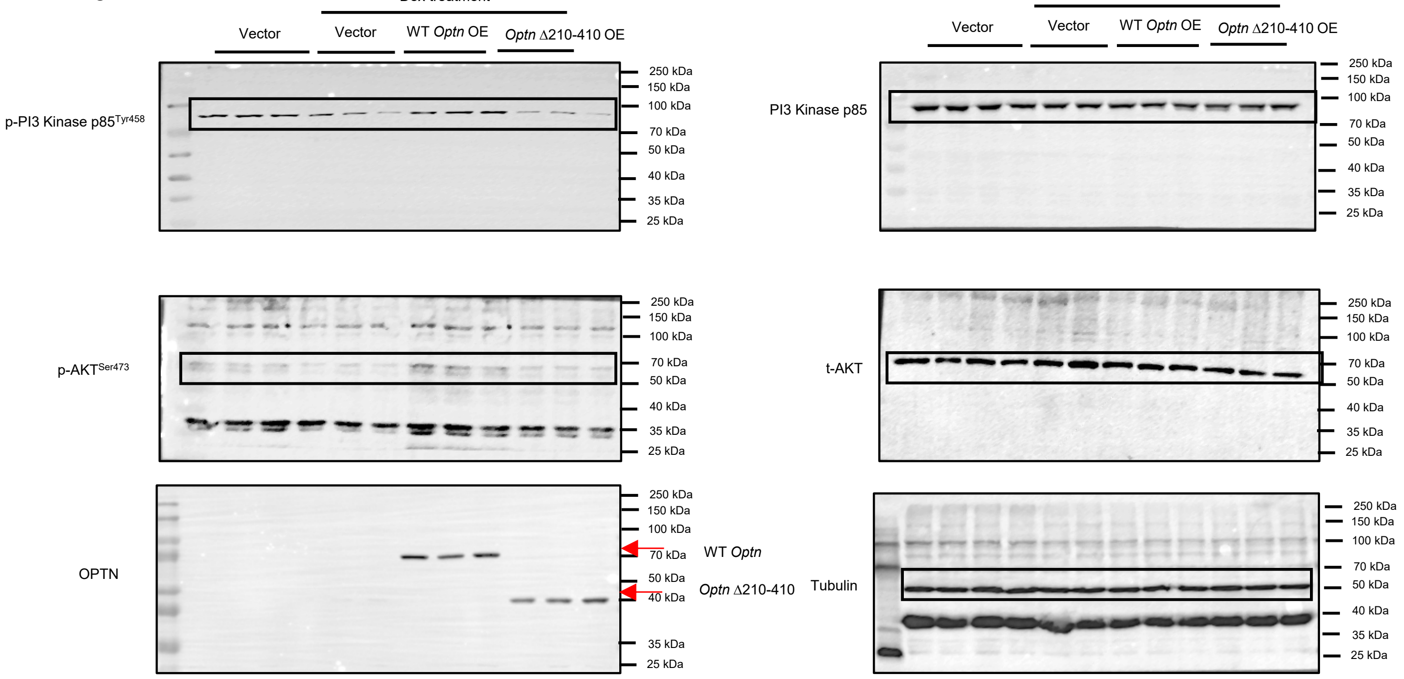

S6C Fig.

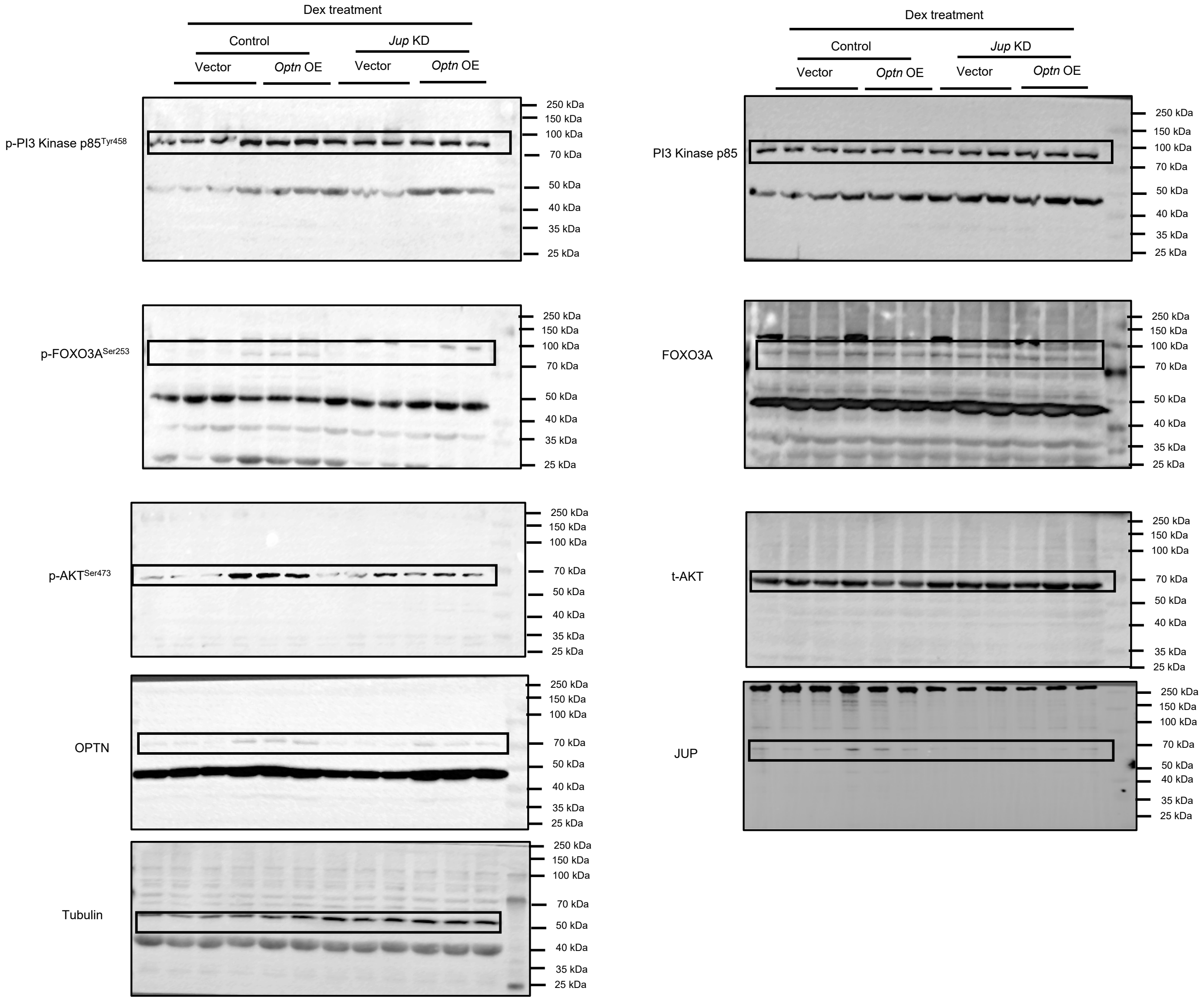

Supplement: S1 Raw Image — (PDF) [file pbio.3003581.s011.pdf]
